# Systemic and immunotoxicity induced by topical application of perfluoroheptane sulfonic acid (PFHpS) or perfluorooctane sulfonic acid (PFOS) in a murine model

**DOI:** 10.1080/1547691X.2024.2371868

**Published:** 2024-07-27

**Authors:** Lisa M. Weatherly, Hillary L. Shane, Laurel G. Jackson, Ewa Lukomska, Rachel Baur, Madison P. Cooper, Stacey E. Anderson

**Affiliations:** Allergy and Clinical Immunology Branch, Health Effects Laboratory Division, National Institute for Occupational Safety and Health, Morgantown, WV, USA

**Keywords:** Perfluoroheptane sulfonic acid (PFHpS;), toxicity, immune, dermal, liver damage, PPAR, immunosuppression

## Abstract

Per- and polyfluoroalkyl substances (PFAS) are a large group of synthetic surfactants of over 12,000 compounds that are incorporated into numerous products for their chemical and physical properties. Studies have associated PFAS with adverse health effects. Although there is a high potential for dermal exposure, these studies are lacking. The present study evaluated the systemic and immunotoxicity of subchronic 28- or 10-days of dermal exposure, respectively, to PFHpS (0.3125–2.5% or 7.82–62.5 mg/kg/dose) or PFOS (0.5% or 12.5 mg/kg/dose) in a murine model. Elevated levels of PFHpS were detected in the serum and urine, suggesting that absorption is occurring through the dermal route. PFHpS induced significantly increased relative liver weight, significantly decreased relative spleen and thymus weight, altered serum chemistries, and altered histopathology. Additionally, PFHpS significantly reduced the humoral immune response and altered immune subsets in the spleen, suggesting immunosuppression. Gene expression changes were observed in the liver, skin, and spleen of genes involved in fatty acid metabolism, necrosis, and inflammation. Immune-cell phenotyping identified significant decreases in B-cells and CD11b^+^ monocyte and/or macrophages in the spleen along with decreases in eosinophils and dendritic cells in the skin. These findings support PFHpS absorption through the skin leading to liver damage and immune suppression.

## Introduction

Per- and polyfluoroalkyl substances (PFAS) are a large group of synthetic surfactants of over 12,000 compounds (United State Environmental Protection Agency [US EPA], 2023), a number that has increased by several thousand due to increased work to identify PFAS-containing products. These compounds are composed of carbon–fluorine bonds that offer chemical and physical properties including thermal, water, and oil resistance (Lau et al. 2007). These compounds are widely integrated into products and processes (Kato et al. 2011) with more than 200 PFAS use categories identified in 2020, include building and construction, electronics, plastic and rubber production, coatings and paints, and lubricants and greases (Glüge et al. 2020). PFAS are also incorporated into food packaging, fire-retardant foams, firefighter personal protective equipment, ski wax, leather, carpets, cosmetics, and personal care products (Kotthoff et al. 2015; Whitehead et al. 2021; United States Food and Drug Administration [US FDA] 2022).

PFAS sulfonic acids are considered long-chain if they contain six or more carbons in their carbon chain. Perfluorooctane sulfonic acid (PFOS) is a widely studied legacy long-chain PFAS containing an eight-carbon structure. US production of PFOS and perfluorooctanoic acid (PFOA) was voluntarily stopped by multiple manufacturers; however, PFOS is still imported and used by companies not participating in the Stewardship program (US EPA 2023). PFOS is still detected in the environment (Kurwadkar et al. 2022), in animals (Death et al. 2021), and in humans (Göckener et al. 2020; Zhao et al. 2022). PFOS has a long half-life of 3.3–27 years in humans (Agency for Toxic Substances and Disease Registry [ATSDR] 2021). Oral PFOS exposure results in liver effects and alterations in immune function in rodents (ATSDR 2021; Ehrlich et al. 2023). Epidemiological studies also show a relationship between PFOS exposure and hepatic, cardiovascular, immune, and developmental effects in humans (ATSDR 2021). The International Agency for Research on Cancer (IARC) recently classified PFOS as a Group 2B possible carcinogen.

As legacy, long-chain PFAS were phased-out; however, alternative PFAS have taken their place. Perfluoroheptane sulfonic acid (PFHpS) is a sulfonic acid PFAS with a seven-carbon chain and is found in outdoor textiles, ski wax, and leather (Kotthoff et al. 2015). PFHpS was the predominant PFAS detected in textile materials at 73.8 μg/kg compared to detected PFOS levels of 3.2 μg/kg (Bečanová et al. 2016). PFHpS has been identified in sludge samples at a mean concentration of 1.98 ng/L, having a lower concentration than PFOS (41.4 ng/L) but higher than PFHxS (0.01 ng/L) (Campo et al. 2014). In river samples in Germany, PFHpS was the second highest detected PFAS species (after PFOA) (Nxumalo et al. 2023). In groundwater samples in China, PFHpS was a main pollutant behind PFOA, with mean concentrations of 51 and 177.33 ng/L, respectively (Li et al. 2022). Norwegian studies detected PFHpS in 100% of the human serum, plasma and whole blood samples (Thepaut et al. 2021), and an increasing trend in PFHpS human blood concentration was observed between 2007/2008 and 2013/2014 (Poothong et al. 2017). In a study of Australian firefighters, the half-life of PFHpS was estimated to be 7.4 years - longer than both PFOS (6.5 years) and PFOA (5.0 years) in this study (Nilsson et al. 2022a).

There is potential for dermal exposure to PFAS *via* commercial products through both occupational, and general environmental contact. Workers involved in the manufacture of PFAS and/or PFAS containing material along with workers in the Public Safety Sector (firefighters and support services, first responders, law enforcement) have a high PFAS exposure risk through the use of and contact with PFAS-containing products, including firefighting foams (Trowbridge et al. 2020; US EPA 2022). Manufacturing workers and firefighters have significantly higher PFAS serum concentrations compared to the general public (Olsen et al. 2003; Khalil et al. 2020). Consumers are also exposed to PFAS through numerous products, through which dermal exposure could occur (Kotthoff et al. 2015). Due to their persistence and lack of degradation, PFAS is detected in the environment (Wang et al. 2017), drinking water, groundwater, and in wastewater treatment plants (Munoz et al. 2017; Lenka et al. 2021; McDonough et al. 2021).

This laboratory previously showed that perfluorobutanoic acid (PFBA), perfluoro-pentanoic acid (PFPeA), perfluorohexanoic acid (PFHxA), perfluoroheptanoic acid (PFHpA), and PFOA were absorbed through the skin and result in systemic toxicity leading to functional and/or altered immune effects in mice (Shane et al. 2020; Weatherly et al. 2021, 2023). Although there is the potential for dermal PFAS exposure, this area of research is lacking. The present study sought to investigate systemic effects that might develop in a murine model after exposure to PFHpS and PFOS. These studies are needed to help fill in knowledge gaps and the results will help with furthering our understanding of the health effects associated with dermal PFAS exposure.

## Materials and methods

### Animals

Female B_6_C_3_F_1_ mice (7–8 wk old) were purchased from The Jackson Laboratory (Bar Harbor, ME) as this is the preferred strain of the National Toxicology Program for evaluating general toxicity. Animals were housed 5 mice/group in ventilated plastic shoe box cage with hardwood chip bedding, and provided modified NIH-31 6% irradiated rodent diet (Harlan Teklad #7913, St. Louis, MO) and sterile tap water from water bottles *ad libitum*. Facility temperature was maintained at 65–78 °F and relative humidity at 30–70%; a light/dark cycle was maintained at 12-hr intervals. All experiments were performed in the AAALAC International accredited National Institute for Occupational Safety and Health (NIOSH) animal facility in accordance with an animal protocol approved by the CDC-Morgantown Institutional Animal Care and Use Committee. This activity was reviewed by CDC, deemed research not involving human subjects and was conducted consistent with applicable federal law and CDC policy.

### Test articles and chemicals

Acetone [CAS #67–64-1] was purchased from Sigma (St. Louis, MO). Perfluoroheptane sulfonic acid (≤ 100%, PFHpS) [CAS #375–92-8] and perfluorooctane sulfonic acid (97%, PFOS) [CAS #1763–23-1] were purchased from Synquest Laboratories (Alachua, FL). PFHpS concentrations were selected based on a range finding study and previous studies with PFAS (Shane et al. 2020; Weatherly et al. 2023).

### PFAS exposures

To evaluate potential systemic and immunotoxic effects, mice (*n* = 5/group) were topically treated on the dorsal surface of each ear (25 μl/ear) with vehicle (acetone), PFHpS (0.3125, 0.625, 1.25% w/v), or PFOS (0.5% w/v) once a day for 28 days. Body weights were measured before exposure and weekly to ensure no body weight change was associated with exposure. Animals were euthanized by CO_2_ asphyxiation ≈24 h after the final exposure.

For immune function studies, mice (*n* = 5/group) were topically treated similarly with vehicle (acetone), PFHpS (0.625, 1.25, 2.5% [w/v]), or PFOS (0.5%) once a day for 10 days. An increased PFHpS concentration was used in these studies as no trends in weight loss were observed by Day 10 (data not shown). Four days prior to euthanasia, the mice were immunized intravenously (IV) with 7.5 × 10^7^ sheep red blood cells (SRBC from single donor animal; Lampire Laboratories, Pipersville, PA) in a 200-μl volume. Again, mice were euthanized by CO_2_ asphyxiation ≈ 24 hr after the final exposure.

### Tissue processing

Following euthanasia, animals were weighed, and blood samples were collected *via* cardiac puncture; thereafter, their liver, spleen, kidneys, and thymus were removed, cleaned of connective tissue, and weighed. Spleen (1/2), draining lymph nodes (dLN) (2 nodes/animal), and ear (1) pinna single cell suspensions were prepared for immune phenotyping by flow cytometry as previously described (Weatherly et al. 2021). Half of one ear pinna and a small lobe of the liver (caudate) were collected and stored in 0.5 ml RNAlater (ThermoFisher Scientific, Waltham, MA) for subsequent gene expression analysis. The remainder of the liver, spleen (1/2), ear pinna (1/2), and one kidney (right) were placed in 10% formalin for later histopathology analyses (see below).

### Serum chemistries

Collected blood samples were transferred to serum separation tubes, and serum isolated from platelets/cells by centrifugation. The material was frozen at −20 °C for subsequent serum chemistry analysis using a Catalyst DX Chemistry Analyzer (IDEXX Laboratories, Westbrook, ME). Endpoints analyzed included: alkaline phosphates (ALKP), urea nitrogen (BUN), glucose (GLU), cholesterol (CHOL), alanine transaminase (ALT), total protein (TP), albumin (ALB), and globulin (GLOB).

### Analytical PFAS detection

Serum collected from each animal and urine pooled for each group of mice were analyzed for PFHpS by Vista Analytical Laboratory (El Dorado Hills, CA), adhering to their standard operating procedures of solid phase extraction and liquid chromatography/tandem mass spectrometry (LC/MS/MS) as previously described (Shoemaker et al. 2008). Quantified PFHpS included the linear isomer only while quantified PFOS included both linear and branched isomers. The initial calibration and continuing calibration verifications met the acceptance criteria as described in Shoemaker et al. (2008). No analytes were detected in the method blank above the reporting limit (200 ng/ml). Labeled standard recoveries for all quality controls and samples were within the acceptance criteria as described in Shoemaker et al. (2008).

### Flow cytometry

Flow cytometry was conducted as described in Weatherly et al. (2023). Data was acquired on a LSR II flow cytometer (BD Biosciences, San Jose, CA) and analyzed using FlowJo software (v.10, TreeStar, Ashland, OR). Cellular populations were defined using the gating strategies outlined in Supplemental Table 1; fluorescence minus ones (FMO) were used as gating controls.

### Gene expression

Gene expressions (listed in Supplemental Table 2) in the isolated tissue samples were evaluated as previously described in Weatherly et al. (2023). Relative-fold gene expression changes (2^−ΔΔCT^) were determined compared to vehicle controls after normalization to b-actin (Applied Biosystems, Waltham, MA).

### Histology

Samples that had been fixed in formalin were sectioned to 5-μm and then underwent hematoxylin and eosin (H&E) staining. All samples were then analyzed by a certified pathologist One slide/organ/animal was examined in each of the five animals/group. The extent of changes in the organs was expressed in terms of histopathology grades of Grade 1 (minimal), Grade 2 (mild), Grade 3 (moderate), Grade 4 (marked), or Grade 5 (severe), with the levels denoting respectively any increasing extent of change.

### Spleen IgM response to SRBC

The primary IgM response to SRBC was enumerated using a modified hemolytic plaque assay (Jerne and Nordin 1963; Shane et al. 2020) as described in Anderson et al. (2013). In brief, ali-quots (100 μl) of the splenocyte suspension generated above were diluted 1:30 or 1:120 with HBSS (Hanks’ Balanced Salt Solution), and then mixed with 0.5 ml warm agar/dextran, 25 μl of 1:1 SRBC suspension, and 25 μl of a 1:4 dilution (1-ml lyophilized; diluted in HBSS) guinea pig complement. The mixture was then then poured into a petri dish, covered with a glass slide, and incubated at 37 °C. After 3 hr, plates were checked for plaques, and the latter were enumerated. Two dilutions were done for each mouse. Results were expressed in terms of both specific activity (IgM PFC/10^6^ spleen cells) and total activity (IgM PFC/spleen).

### Serum IgM response to SRBC

Serum samples were analyzed for anti-SRBC IgM using a commercially available ELISA kit (Life Diagnostics, West Chester, PA), following manufacturer recommendations with modifications. In brief, test serum was diluted (1:40, 1:80, 1:160, and 1:320) with kit diluent YD30–1, and incubated in anti-SRBC-coated microtiter plates for 45 min at 25 °C. Optical density was then measured at 450 nm in a Spectra Max-Plus plate reader (Molecular Devices, San Jose, CA). The anti-SRBC IgM concentration in each test sample was determined by comparison to a standard curve generated in parallel using SoftMax Pro software (Molecular Devices, Sunnyvale, CA). All outcomes were reported in terms of units of anti-SRBC IgM (U/ml) plotted vs absorbance values at 450 nm.

### Statistical analysis

Results are expressed as means ± SE from 5 mice/group. A one-way analysis of variance (ANOVA) was conducted on data from the animal studies. If the ANOVA showed significance at *p* ≤ 0.05, a Dunnett’s Multiple Range *t*-test was used to compare treatment groups with the control group. A Kruskal-Wallis with Dunn’s post-test was conducted for gene expression analysis in groups that had unequal variances. Linear trend tests were conducted to show a dose response in select endpoints tested. All analyses were performed using Prism software (v.9.2, GraphPad, San Diego, CA). Significance was designated by *p ≤ 0.05, **p ≤ 0.01, and ***p ≤ 0.001.

## Results

### PFHpS and PFOS induced significant alterations in serum and urine PFAS concentrations after 28-day dermal exposure

**Figure 1.**
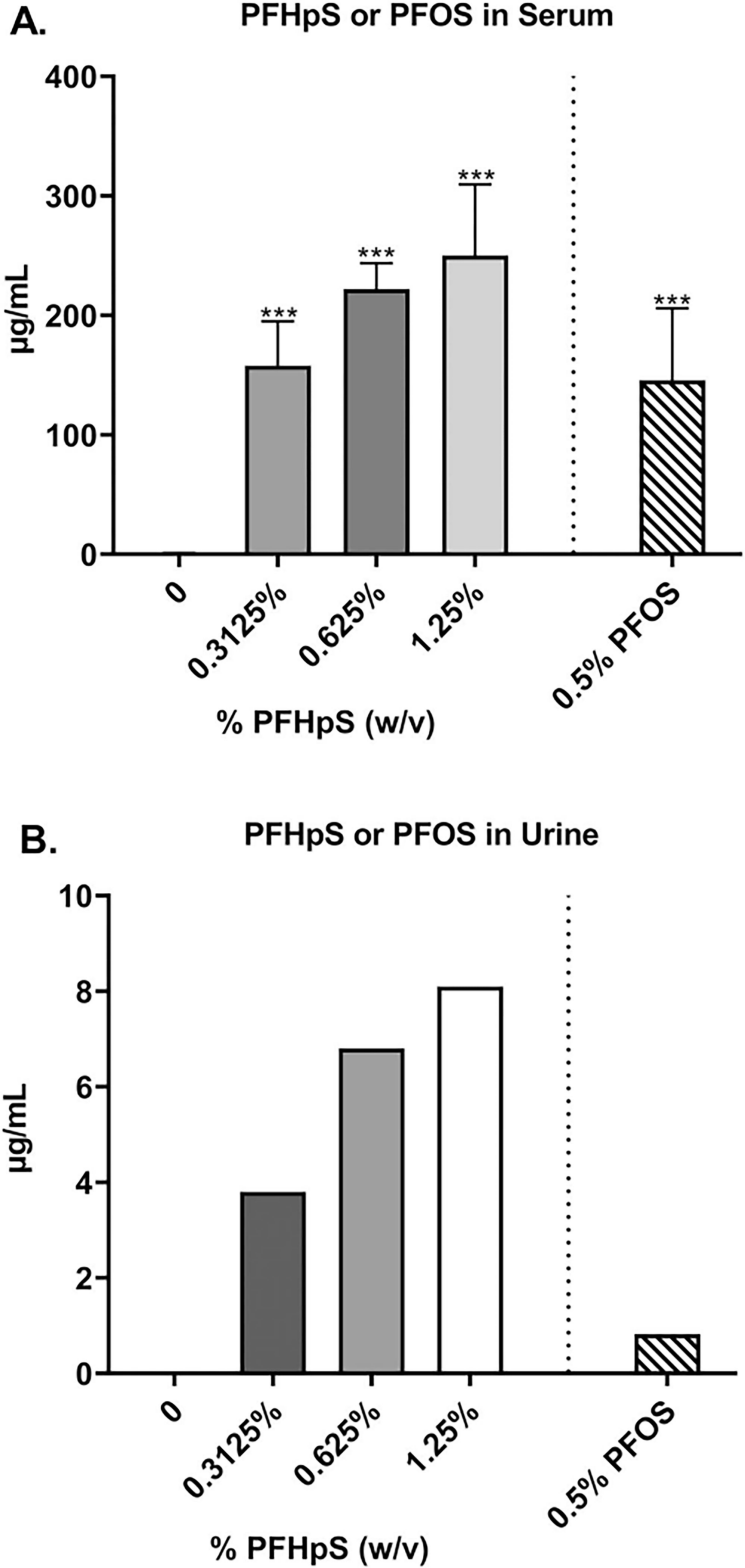
Changes in PFHpS and PFOS concentration in serum and urine after dermal exposure. Analysis of changes in the concentration of PFHpS and PFOS in serum (A) and concentration of PFHpS and PFOS in urine samples (B) following 28 days of PFHpS exposure. Each concentration represents mean (± SE) of 5 mice/group. Urine concentrations are five pooled samples per group. Statistical significance, relative to 0% vehicle control, was determined by one-way ANOVA followed by a Dunnett’s post-test (PFHpS) or a *t*-test (PFOS) indicated as ***p < 0.001.

A significant increase in PFHpS serum concentration was observed with 0.3125, 0.625, and 1.25% PFHpS (Figure 1(A)); levels increased from 0.744 μg/ml (control) to 158, 222, and 250 μg/ml, respectively. This suggested that there was consequential absorption occurring after PFHpS dermal exposure. Statistical analysis could not be performed on the urine samples as each concentration was from five pooled samples with a single data point. Urine PFHpS concentration increased from 0 − 3.8 μg/ml with 0.3125% PFHpS, 6.8 μg/ml with 0.625% PFHpS, and 8.10 μg/ml with 1.25% PFHpS (Figure 1(B)). PFOS also increased in serum (145.6 μg/ml) and urine (0.82 μg/ml) with a significant increase observed in the serum (Figure 1(A and B)).

### PFHpS or PFOS dermal exposure for 28 days resulted in significant altered organ weight

**Figure 2.**
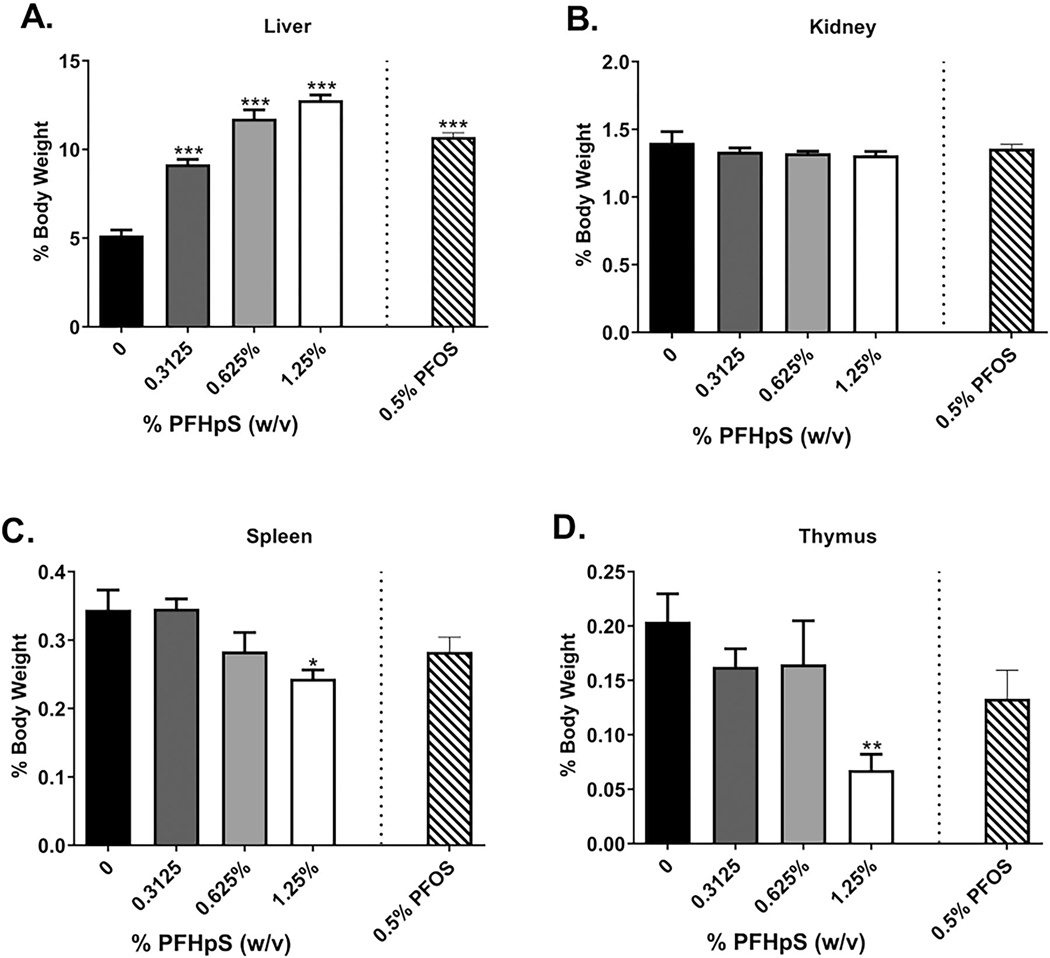
Changes in relative organ weights after dermal exposure to PFHpS or PFOS. Analysis of changes in (A) liver, (B) kidney, (C) spleen, and (D) thymus weights following 28 days of PFHpS exposure. Data displayed as organ weight as % body weight. Each concentration represents mean (± SE) of 5 mice/group. Statistical significance, relative to 0% control, was determined by one-way ANOVA followed by a Dunnett’s post-test (PFHpS) or a *t*-test (PFOS) indicated as *p < 0.05, **p < 0.01, ***p < 0.001.

A significant increase in relative liver weights and decrease in spleen and thymus weights was observed with PFHpS exposure (Figure 2). Relative liver weight significantly increased following exposure to 0.3125, 0.625, and 1.25% PFHpS (78, 128, and 148%, respectively, vs. values for vehicle-treated mice) (Figure 2(A)). Relative spleen weights were significantly decreased by 29% with the highest PFHpS (1.25%) concentration (Figure 2(C)). Relative thymus weight also decreased with 1.25% PFHpS (67%) (Figure 2(D)). No change in relative weight was observed in the kidneys (Figure 2(B)). A significant decrease in change in body weight was observed with 0.625% and 1.25% PFHpS (Supplemental Figure 1); however, the decrease in body weight observed was less than 10%. PFOS (0.5%) was used to compare PFHpS to a legacy long-chain PFAS. PFOS induced an increase in relative liver weights (107%) but no significant change was observed in the kidney, spleen, and thymus weights (Figure 2). PFOS did induce overt toxicity, with body weight decreasing ~ 14% from the start of the study (Supplemental Figure 1). The significant changes in organ weights were dose dependent with PFHpS exposure [significant linear trend *p* < 0.001 (liver) and *p* < 0.01 (spleen and thymus)]. Absolute organ weights not corrected for total body weight are reported in Supplemental Table 3, with significant increases in liver and kidney mass (0.3125, 0.625, 1.25% PFHpS and 0.5% PFOS), significant decreases in spleen mass (0.625, 1.25% PFHpS and 0.5% PFOS), and significant decreases in thymus mass (1.25% PFHpS and 0.5% PFOS).

### Dermal exposure to PFHpS or PFOS alters serum chemistries

**Figure 3.**
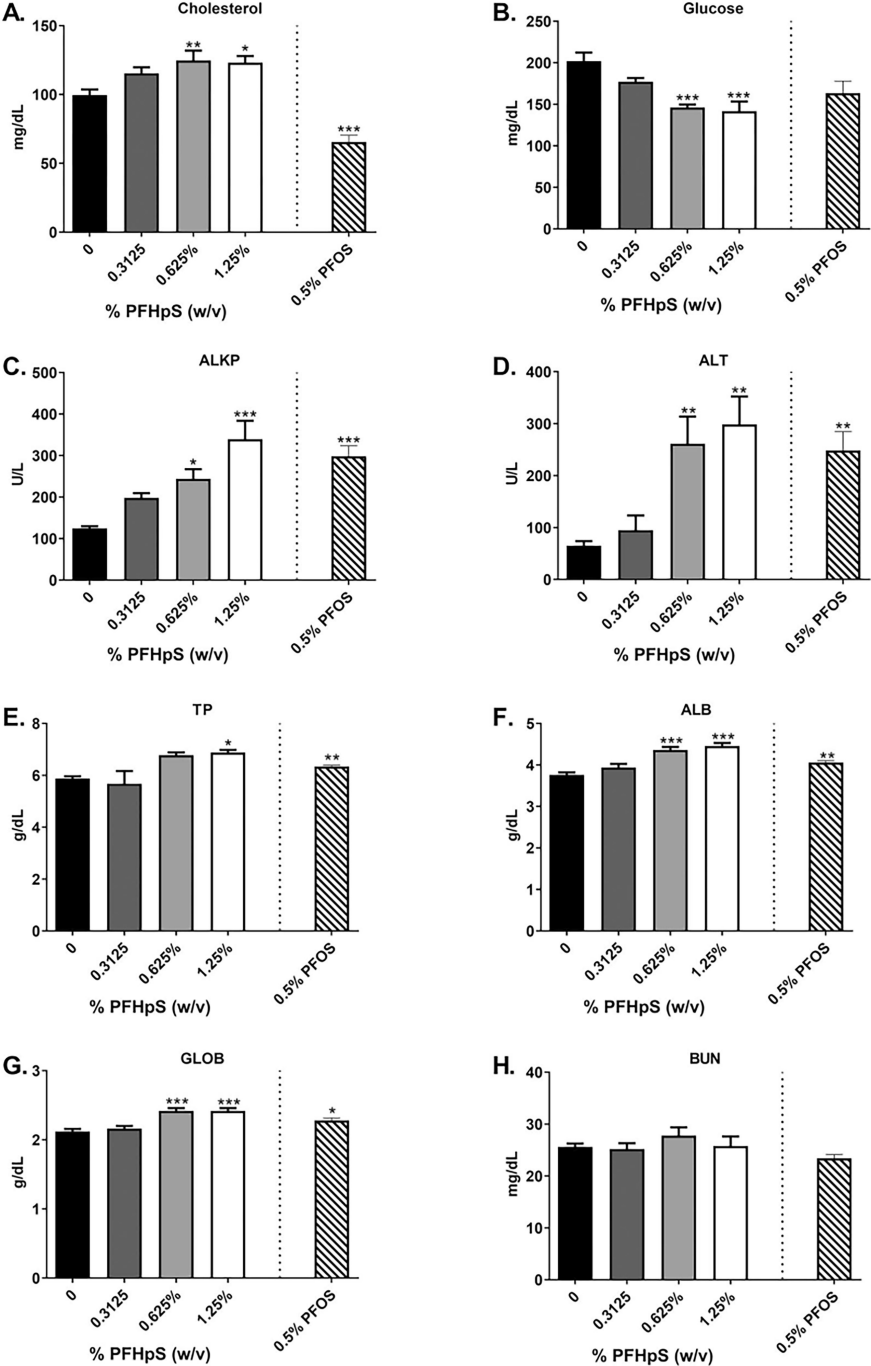
Changes in serum chemistry after dermal exposure to PFHpS or PFOS. Analysis of changes in (A) cholesterol, (B) glucose (C) alkaline phosphatase (ALKP), (D) alanine amino-transferase (ALT), (E) total protein (TP), (F) albumin (ALB), (G) globulin and (H) urea nitrogen following 28 days of PFHpS exposure. Each concentration represents the mean (± SE) of 5 mice/group. Statistical significance, relative to 0% vehicle control, was determined by one-way ANOVA followed by a dunnett’s post-test (PFHpS) or a *t*-test (PFOS) indicated as *p < 0.05, **p < 0.01, ***p < 0.001.

After 28 days of PFHpS exposure, there was a significant increase in serum cholesterol, ALKP, ALT, TP, ALB, and GLOB, and a significant decrease in glucose (Figure 3). Cholesterol, ALKP, ALT, ALB, and GLOB were increased at two PFHpS concentrations with increases of 24, 173, 358, and 14%, respectively, with 1.25% PFHpS (Figures 3(A, C, D, F, and G). TP increased 17% with 1.25% PFHpS (Figure 3(E)) and glucose decreased 28 and 30% with 0.625 and 1.25% PFHpS, respectively (Figure 3(B)). No significant changes were observed in BUN with PFHpS exposure (Figure 3(H)). Cholesterol decreased by 34% and ALKP, ALT, TP, ALB, and GLOB increased by 139.0, 281.0, 7.8, 8.0, and 7.5%, respectively, with 0.5% PFOS (Figure 3). No change was observed with glucose or BUN with 0.5% PFOS exposure. The significant changes in serum chemistries were dose dependent [significant linear trend *p* < 0.001 (glucose, ALKP, ALT, ALB, GLOB), *p* < 0.01 (cholesterol, TP)].

### 28-day dermal exposure of PFHpS or PFOS results in histopathological changes in liver, skin, and spleen

**Figure 4.**
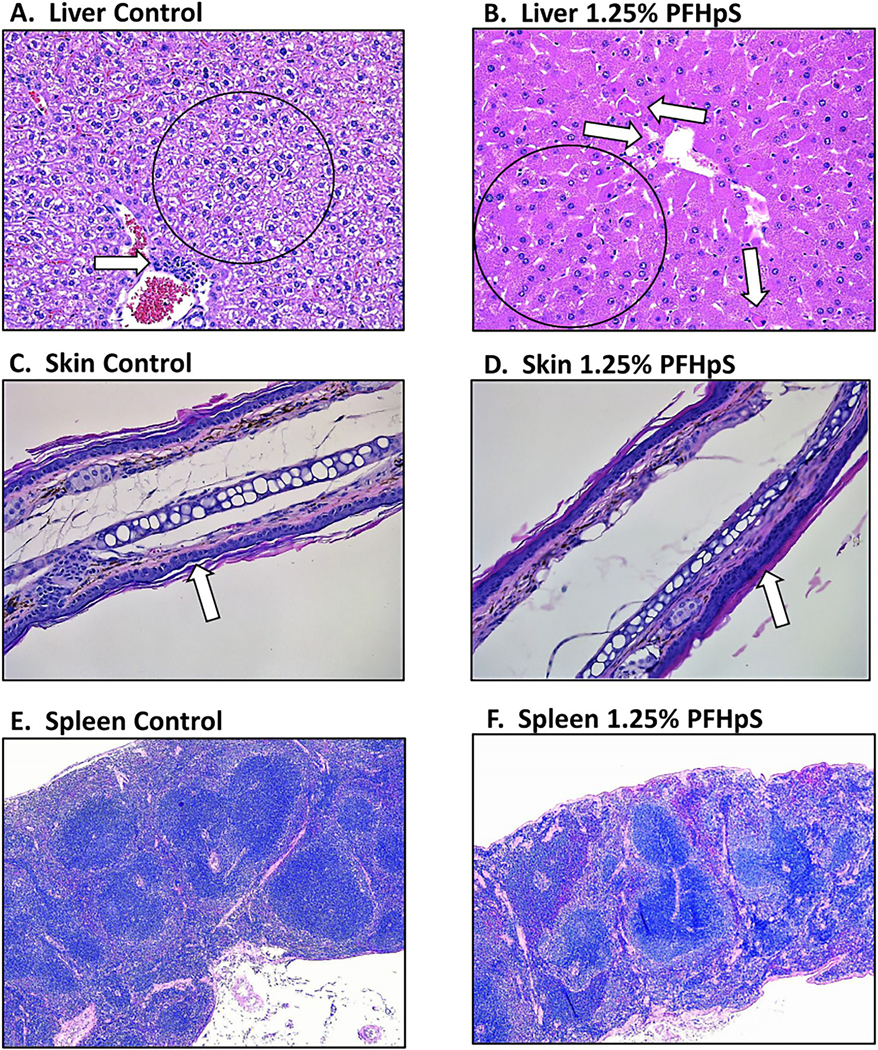
Histopathology of liver, ear, and spleen following dermal exposure to PFHpS. Representative H&E-stained liver, ear, and spleen sections from control and 1.25% PFHpS-treated mice. Vehicle control 0% PFHpS exposure shows normal liver with mononuclear cell infiltrate (arrow), note size of hepatocytes and normal cytoplasmic rarefaction (black circle), 20× magnification (a). Marked hepatocyte hypertrophy (note size and eosinophilia of hepatocytes (black circle)) and minimal multifocal necrosis of hepatocytes (arrows) with a few associated neutrophils (inflammation) was found in 1.25% PFHpS exposed mice, 20× magnification (B). Vehicle control 0% PFHpS exposure shows normal ear (skin), note thickness of epidermis is 1–2 cell layers (arrow), 20× magnification (C). Minimal epidermal hyperplasia was observed in 1.25% PFHpS exposed mice with thickness of epidermis 3–4 cell layers (arrow), 20× magnification (D). Vehicle control 0% PFHpS exposure shows normal spleen (note overall thickness of spleen and size of lymphoid aggregates), 5× magnification (E). Mildly decreased lymphocytic cellularity, decreased overall thickness of the spleen, and decreased size of lymphoid aggregates were observed with 1.25% PFHpS exposed mice, 5× magnification (F).

The majority of histopathological alterations were observed in the liver; examination revealed PFHpS-induced hepatocellular hypertrophy in all exposed animals (Table 1). The greatest severity was observed at 1.25% PFHpS with all five animals exhibiting marked hyper-trophy compared to vehicle-exposed animals (Figure 4(A and B)). Hepatocellular hypertrophy was characterized by increased cytoplasmic eosinophilia, decreased glycogen content, and increased hepatocyte volume predominantly in centrilobular locations, but in animals with marked hypertrophy, the entire hepatic plate was affected. Hepatocyte necrosis typically affected single cells scattered within the parenchyma, with 5/5 mice in both 0.625 and 1.25% PFHpS exposure groups evincing minimal necrosis. Affected small groups of hepatocytes in 2/5 animals given 0.625% PFHpS and 1/5 animals that received 1.25% PFHpS associated with neutrophilic inflammation. Hepatocyte necrosis was considered an adverse change. Control vehicle exposure resulted in a normal liver with mononuclear cell infiltrates (Figure 4(A)), compared to a decreased incidence of mononuclear cell infiltrates in the liver with 1.25% PFHpS (Table 1). PFOS also gave rise to moderate hypertrophy (5/5), necrosis (4/5), and a decrease in mononuclear cell infiltrates (2/5).

At the site of exposure, dose-related epidermal hyperplasia, or an increased number of keratinocyte layers, was observed at doses ≥ 0.625% PFHpS while control exposure showed normal epidermal thickness (Figure 4(C and D); Table 1). In the spleen, controls displayed normal overall thickness and lymphoid aggregate size, while 1.25% PFHpS exposure decreased overall spleen thickness and lymphoid aggregate size, and increased the incidence of minimally decreased lymphocyte cellularity (4/5 mice) (Table 1; Figure 4(E and F)). Decreased cellularity was characterized by decreased lymphocytes within the spleen resulting in a smaller cross-sectional area. PFOS exposure showed no histopathological changes on the skin at site of exposure and in the spleen induced a decrease in cellularity in 3/5 mice (Table 1).

### Dermal PFHpS or PFOS exposure results in changes in liver and skin gene expression

**Figure 5.**
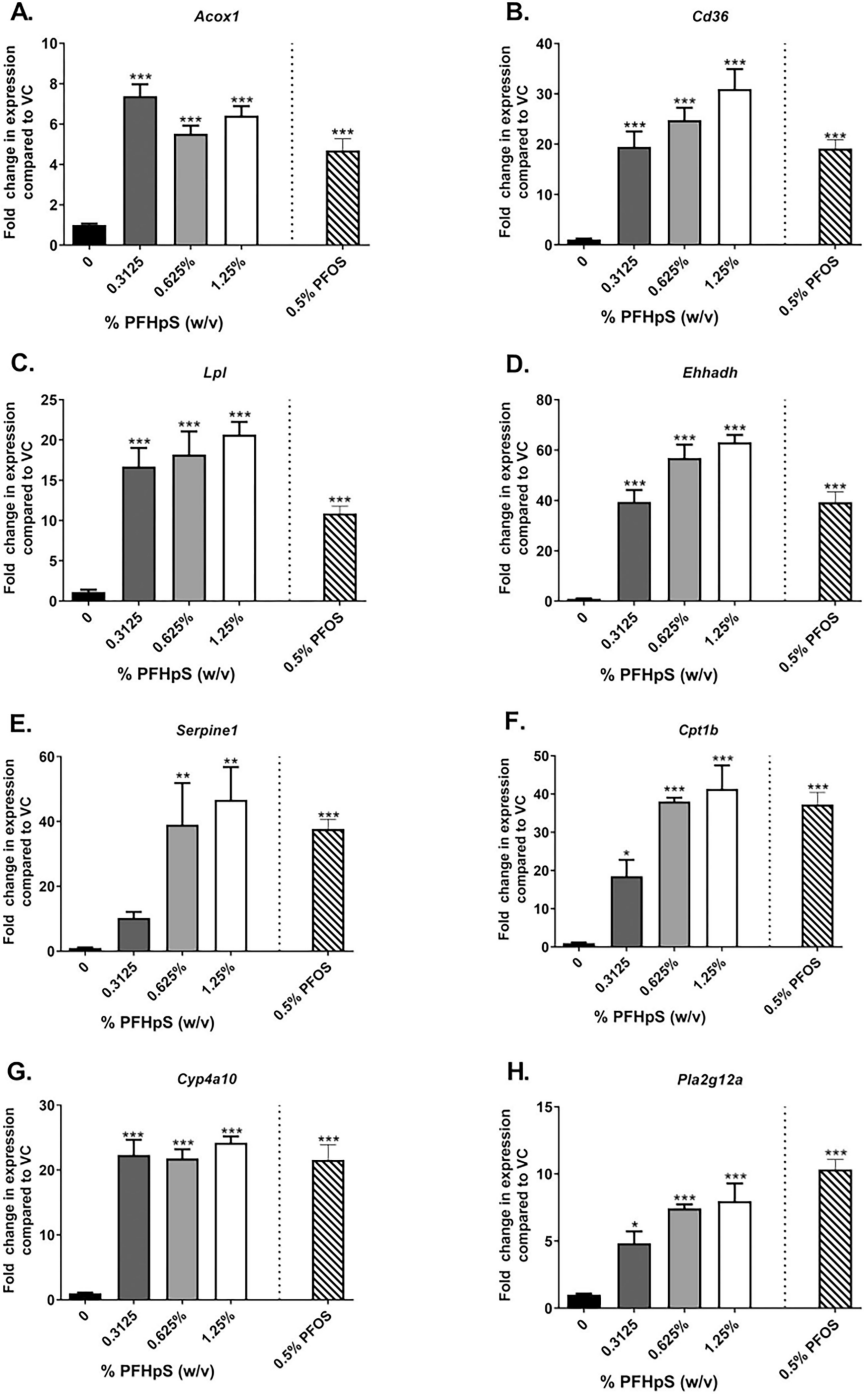

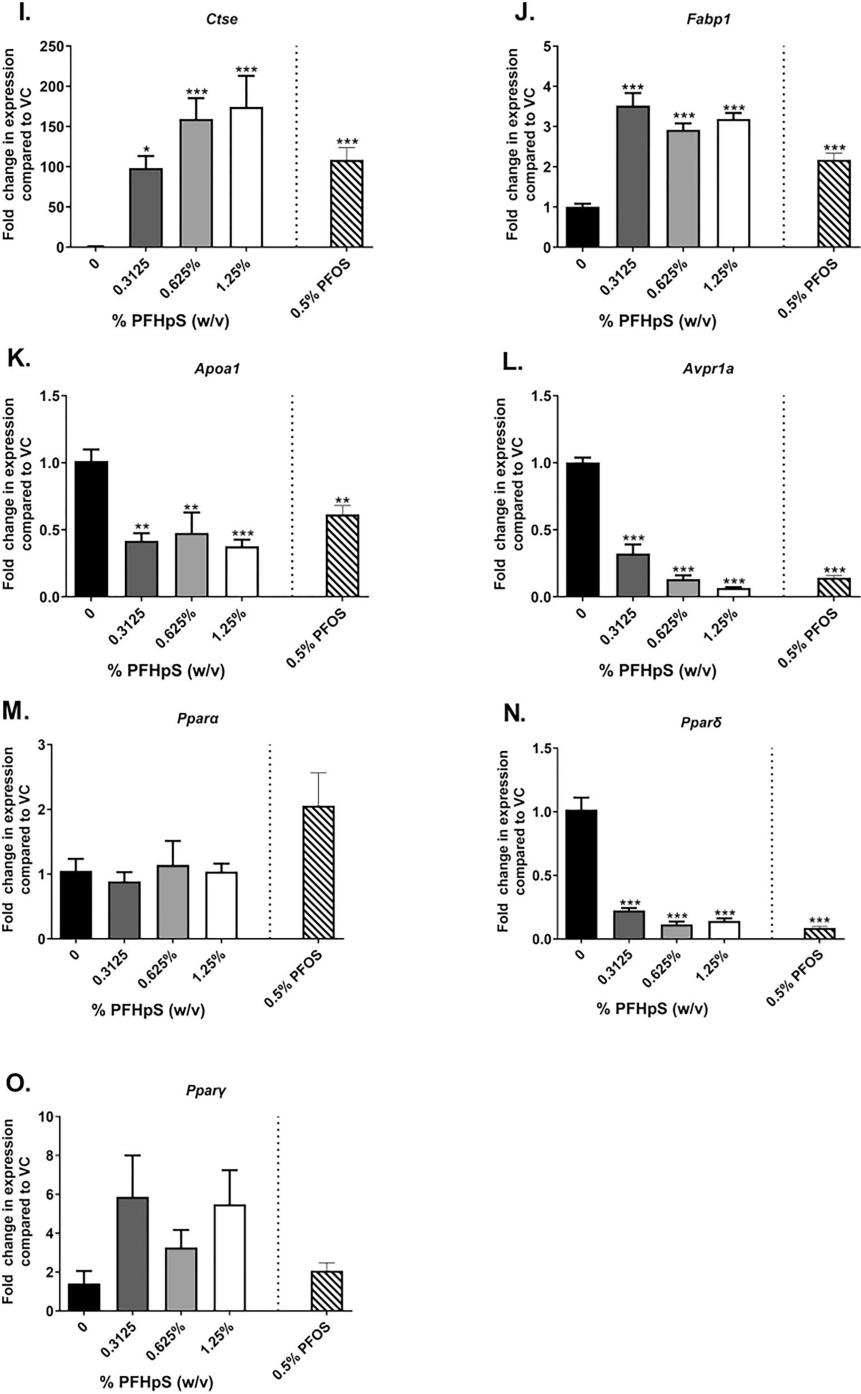
Liver gene expression following dermal exposure to PFHpS. Gene expression in the liver following 28 days of PFHpS exposure. Changes in (A) *Acox1*, (B) *Cd36*, (C) *Lpl*, (D) *Ehhadh*, (E) *Serpine1*, (F) *Cpt1b*, (G) *Cyp4a10*, (H) *Pla2g12a*, (I) *Cset*, (J) *Fabp1*, (K) *Apoa1*, (L) *Avpr1a*, (M) *Pparα*, (N) *Pparδ*, and (O) *Pparγ* were evaluated. Data shown are means (± SE) of 5 mice/group. Statistical significance, relative to 0% vehicle control (VC), was determined by one-way ANOVA with a dunnett’s post-test (PFHpS) or a *t*-test (PFOS) where *p < 0.05, **p < 0.01, ***p < 0.001. Kruskal-wallis with dunn’s post-test was conducted for *Serpine1 and pparγ* due to unequal variance.

To further investigate the mechanism of PFHpS systemic toxicity, gene expression in the liver was investigated based on previous PCR pathway-based arrays (Weatherly et al. 2021). Genes involved in steatosis (*Cd36, Lpl*), hepatoxicity (*Avpr1a, Pla2g12a*), necrosis (*Serpine1*), fatty acid metabolism (*Acox1, Cpt1b, Cyp4a10, Ehhadh*), lipid transport (*Apoa1*), and PPAR transcription factors (*Pparδ*) were altered with PFHpS exposure compared to vehicle-exposed animals (Figure 5). Increases in *Acox1, Cd36, Lpl, Ehhadh, Cpt1b, Cyp4a10, Pla2g12a, Ctse, and Fabp1* gene expression were observed at all three PFHpS concentrations with 6-, 31-, 21-, 63-, 41-, 24-, 8-, 175-, and 3-fold increases at 1.25% PFHpS, respectively (Figure 5(A–D, F–H)). A large increase was also seen in *Serpine1* (1.25%, 47-fold) (Figure 5(E)). Decreases in *Apoa1*, *Avpr*, and *Pparδ* expression was observed with all three PFHpS exposures (Figure 5(K, L, N)). No significant change was observed in either *Pparα* or *Pparγ* (Figure 5(M and O)). PFOS (0.5%) increased *Acox1, Cd36, Lpl, Ehhadh, Serpine1, Cpt1b, Cyp4a10, Pla2g12a, Ctse, and Fabp1* and decreased *Apoa1, Avpr1a,* and *Pparδ* to a similar level to PFHpS exposure. The significant changes in liver gene expression were dose dependent [significant linear trend *p* < 0.001 (*Acox1, Cd36, Lpl, Ehhadh, Serpine1, Cpt1b, Cyp4a10, Pla2g12a, Ctse, Fabp1, Apoa1, Avpr1a, Pparδ*)].

**Figure 6.**
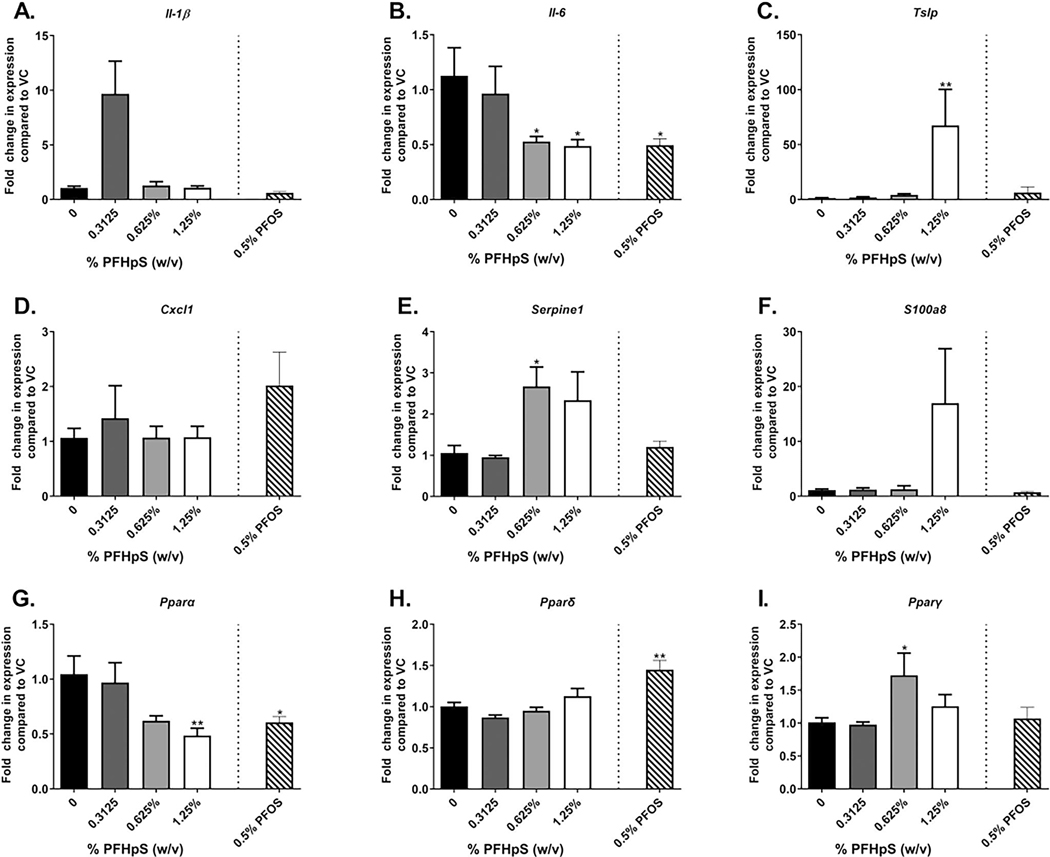
Skin gene expression following dermal exposure to PFHpS or PFOS. Gene expression in the skin following 28 days of PFHpS exposure. Changes in (A) *Il-1β*, (B) *Il-6*, (C) *Tslp*, (D) *Cxcl1*, (E) *Serpine1*, (F) *S100a8*, (G) *Pparα*, (H) *Pparδ*, and (I) *Pparγ* were evaluated. Data shown are means (± SE) of 4–5 mice/group. Statistical significance, relative to 0% vehicle control (VC), was determined by one-way ANOVA with a Dunnett’s post-test (PFHpS) or a *t*-test (PFOS) where *p < 0.05, **p < 0.01, ***p < 0.001. Kruskal–wallis with a Dunn’s post-test was conducted for *Il-1β*, *tslp*, *Serpine1, S100a8,* and *pparα* due to unequal variance.

**Figure 7.**
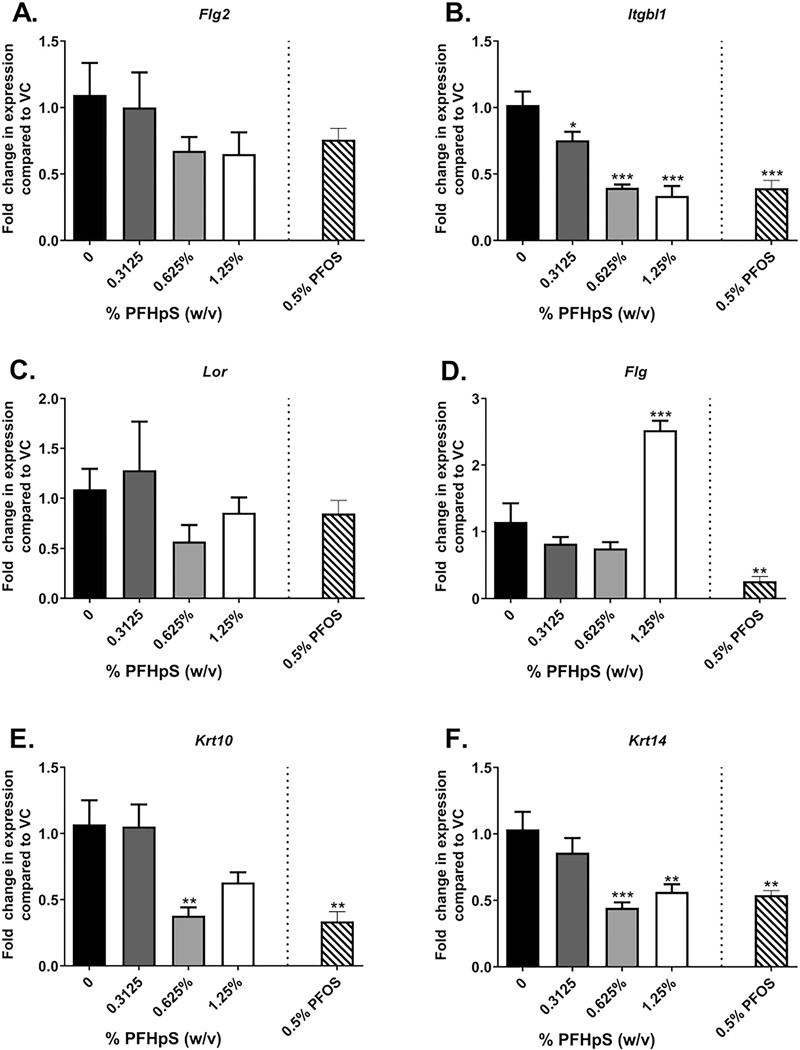
Skin barrier gene expression following dermal exposure to PFHpS or PFOS. Gene expression in the skin following 28 days of PFHpS exposure. Changes in (A) *Flg2*, (B) *Itgbl1*, (C) *Lor*, (D) *Flg*, (E) *Krt10*, and (F) *Krt14* were evaluated. Data shown are means (± SE) of 5 mice/group. Statistical significance relative to 0% vehicle control (VC) was determined by one-way ANOVA with a dunnett’s post-test (PFHpS) or a *t*-test (PFOS) where *p < 0.05, **p < 0.01, ***p < 0.001.

Skin gene expression analysis was conducted to help define the mechanism of PFHpS dermal toxicity. Inflammatory cytokine *Il-6* decreased with 0.625 and 1.25% PFHpS exposure compared to vehicle-exposed animals (Figure 6(B)); no significant changes were observed with *Il-1β* (Figure 6(A)). Expressions of *Tslp* (67-fold) (T_H_2-skewing cytokine) and *Serpine1* (2.7-fold) (involved in necrosis) increased with 1.25 and 0.625% PFHpS, respectively (Figure 6(C and E)). In the skin, *Pparα* gene expression decreased with 1.25% PFHpS, but an increase was seen in *Pparγ* with 0.625% and no change was observed with *Pparδ* (Figure 6(G–I)). Four genes involved in the skin barrier were altered. An increase in *Flg* gene expression with 1.25% PFHpS was observed while *Itgbl1* (0.3125–1.25%), *Krt10* (0.625%) and *Krt14* (0.625–1.25%) decreased after 28 days of PFHpS exposure (Figure 7(B, D–F)). No changes were seen in the skin barrier genes *Flg2* and *Lor*. PFOS (0.5%) decreased *Il-6, Pparα, Itgbl1, Krt10*, and *Krt14* and increased *Pparδ* gene expression (Figures 6(B, G, and H) and 7(B, E, and F). The significant changes in skin gene expression were dose-dependent [significant linear trend *p<* 0.001 (*Itgbl1, Krt14*), *p*< 0.01 (*Il-6, Pparα, Krt10*), *p*< 0.05 (*Tslp, Serpine1*)].

### Dermal exposure of PFHpS or PFOS for 28 days resulted in significant phenotypic changes in skin and dLN

**Figure 8.**
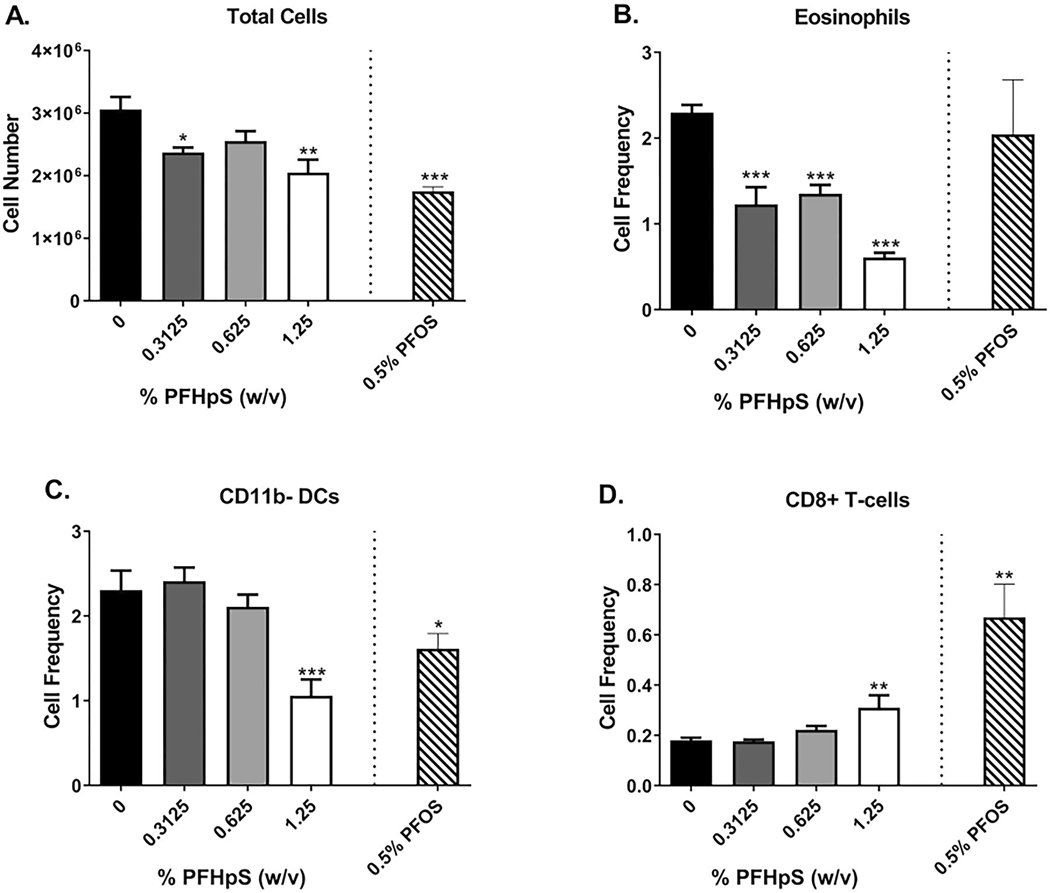
Skin phenotyping following dermal exposure to PFHpS. Phenotyping in the ear following 28 days of PFHpS exposure. Changes in total (A) cells, (B) eosinophils, (C) CD11b^−^ DC, and (D) CD8^+^ T-cells were evaluated and quantified *via* flow cytometry. Statistical significance relative to 0% vehicle control (VC) was determined by one-way ANOVA with a Dunnett’s post-test (PFHpS) or a *t*-test (PFOS) where *p < 0.05, **p < 0.01, ***p < 0.001.

Overall, phenotypic analysis of the ear pinna following 28 days of PFHpS exposure resulted in a decrease in total cellularity (Figure 8(A)). Decreases in the number and frequency of eosinophils (0.3125, 0.625, 1.25%) and CD11b^−^ DC (1.25%) were seen after PFHpS exposure (Figure 8(B and C); Supplemental Table 4). An increase in cell frequency of CD8^+^ cells and of neutrophils occurred with 1.25% PFHpS (Figure 8(D)). The significant changes in skin cellularity were dose dependent [significant linear trend *p* < 0.001 (eosinophil cell number and frequency, CD11b^−^ DC cell number and frequency), *p* < 0.01 (total cell number, CD8^+^ cell frequency, neutrophil cell frequency)]. PFOS (0.5%) induced decreases in total cellularity, CD45^+^, CD4^+^, eosinophil, and CD11b^+^ DC cell number, decreases in both number and frequency of NK cells and CDC11b^−^ DC, and increased CD8^+^ cell frequency (Supplemental Table 4).

Phenotypic changes were also observed in the dLN, showing a significant increase in CD8^+^ T-cell number with 0.625 and 1.25% PFHpS (Supplemental Table 5). A decrease in B-cell frequency was observed with 0.625% PFHpS along with an increase in both number and frequency of dendritic cells with 1.25% PFHpS. Changes in mean fluorescence intensity (MFI) of MHC-II and CD86 on B-cells and DC (1.25%) was also observed with PFHpS exposure (Supplemental Table 4). PFOS (0.5%) showed fewer changes with increases seen in eosinophil and DC frequency (Supplemental Table 5).

### Dermal PFHpS or PFOS exposure resulted in significant changes in spleen phenotyping and gene expression

**Figure 9.**
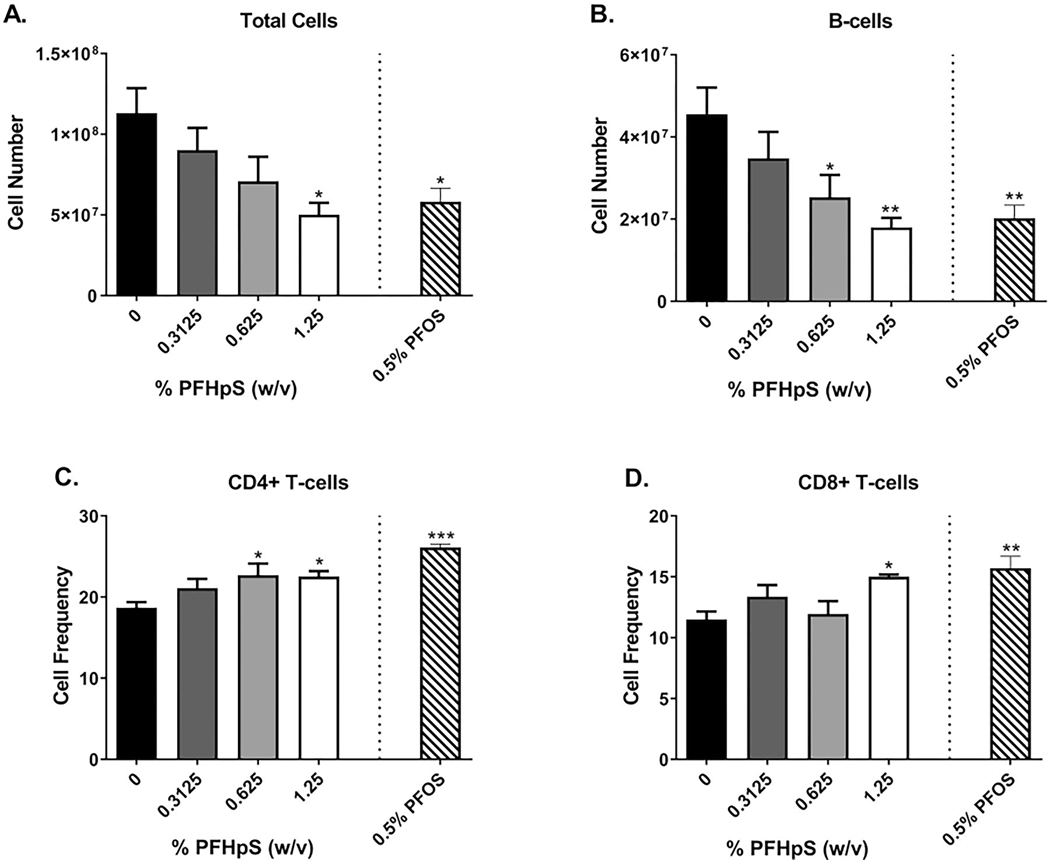
Spleen phenotyping following dermal exposure to PFHpS. Phenotyping in the spleen following 28 days of PFHpS exposure. Changes in total (A) cells, (B) B-cells, (C) CD4^+^ T-cells, and (D) CD8^+^ T-cells were evaluated and quantified via flow cytometry. Statistical significance, relative to 0% vehicle control (VC), was determined by one-way ANOVA with a Dunnett’s post-test (PFHpS) or a *t*-test (PFOS) where *p < 0.05, **p < 0.01, ***p < 0.001.

Consistent with the decrease in organ weight, PFHpS dermal exposure induced a decrease in total cellularity after 28 days of exposure in the spleen with 1.25% PFHpS compared to vehicle-exposed animals (Figure 9(A); Supplemental Table 6). An increase in frequency of CD4^+^ (0.625, 1.25%) and CD8^+^ (1.25%) T-cells occurred with PFHpS exposure (Figure 9(C and D)). PFHpS also induced a decrease in B-cell number (0.625, 1.25%) (Figure 9(B)). CD11b^+^ cells decreased in both cell number and frequency, and CD11b^+^Ly6c^−^ cell number was decreased. MFI of MHC-II increased on B-cells (1.25%), and MFI of CD86 increased on B-cells (0.3125%) and decreased on DC (0.625%) (Supplemental Table 6). The significant changes in spleen gene expression were dose-dependent [significant linear trend *p* < 0.001 (CD11b^+^ cell number, CD11b^+^Ly6C^−^ cell number), *p* < 0.01 (total cell number, B-cell number, MFI of MHC-II on B-cells), *p* < 0.05 (CD4^+^ and CD8^+^ T cell frequency, CD11b^+^ cell frequency]. PFOS (0.5%) decreased total cellularity, B-cell and dendritic cell number and frequency, neutrophil, CD11b^+^, CD11b^+^Ly6C^+^, and CD11b^−^Ly6C^−^ cell number and increased CD4^+^ and CD8^+^ T cell frequency (Supplemental Table 6).

**Figure 10.**
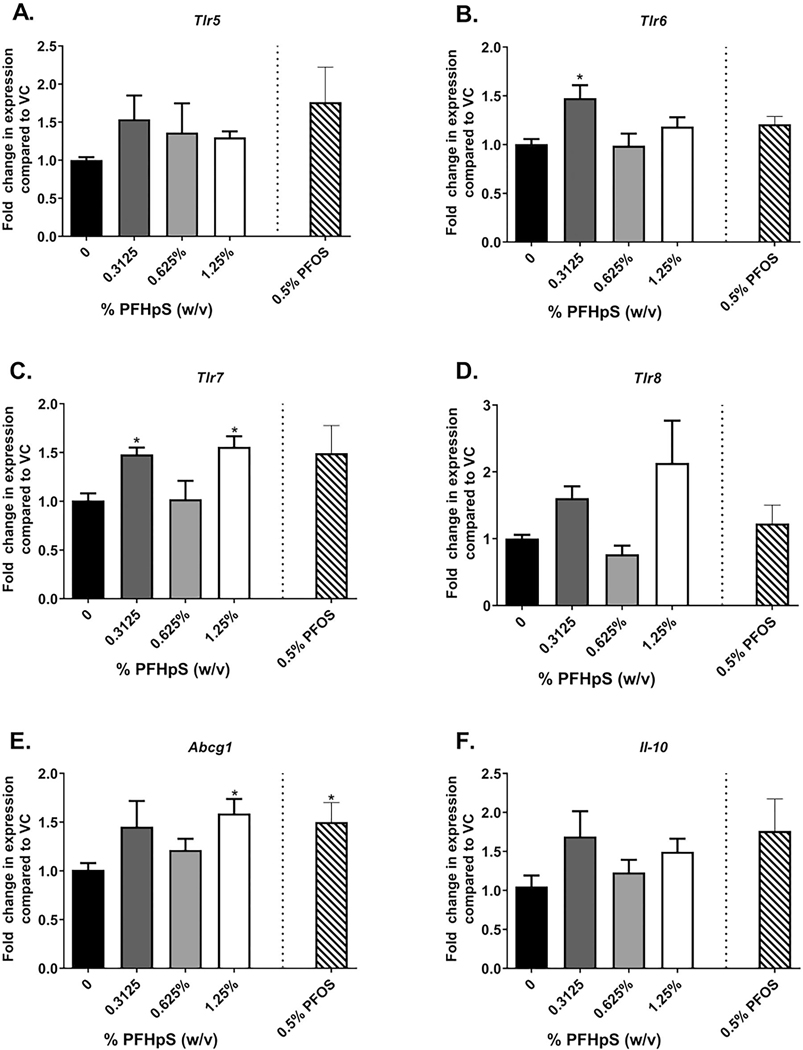
Spleen gene expression following dermal exposure to PFHpS or PFOS. Gene expression in the spleen following 28 days of PFHpS exposure. Changes in (A) *Tlr5*, (B) *Tlr6*, (C) *Tlr7*, (D) *Tlr8*, (E) *Abcg1*, and (F) *Il-10* were evaluated. Data shown are means (± SE) of 5 mice/group. Statistical significance, relative to 0% vehicle control (VC), was determined by one-way ANOVA with Dunnett’s post-test (PFHpS) or a *t*-test (PFOS) where *p < 0.05, **p < 0.01, ***p < 0.001. Kruskal-wallis with Dunn’s post-test was conducted for *Tlr5*, *Tlr8*, and *Abcg1* due to unequal variance.

To further investigate immunotoxicity in the spleen, gene expression was also evaluated. Genes were chosen based on previous studies with PFAS investigating two PCR pathway-based arrays (Immunotoxicity and Innate and Adaptive Immune Responses arrays). The expression of some genes involved in immunotoxicity (*Abcg1)* and innate immunity (*Tlr6, Tlr7*) were seen to have been altered (Figure 10). *Tlr6, Tlr7, and Abcg1* expression minimally but significantly increased with some PFHpS exposures (Figure 10(B, C, and E)), while PFOS (0.5%) minimally increased *Abcg1* gene expression (Figure 10(E)).

### Dermal exposure of PFHpS or PFOS suppressed the humoral immune response

**Figure 11.**
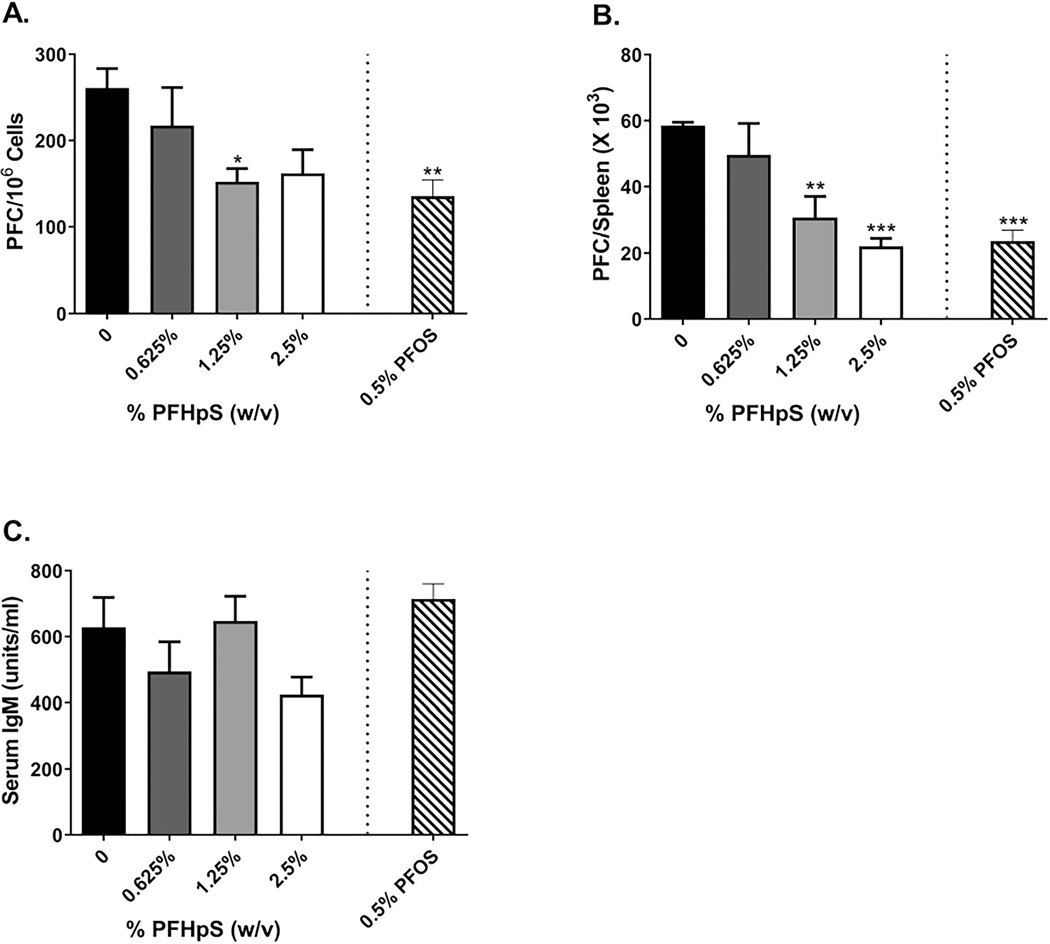
Dermal PFHpS and PFOS exposure suppresses the spleen IgM response to SRBC. Analysis of antibody producing spleen cells after a 10-day dermal exposure to PFHpS suppressed the (A) specific activity and (B) total activity, but not (C) serum IgM response to SRBC. Bars shown are mean fold-change (± SE) of 4–5 mice/group. Statistical significance, relative to 0% vehicle control, was determined by one-way ANOVA followed by Dunnett’s post-test (PFHpS) or a *t*-test (PFOS) indicated as *p < 0.05, **p < 0.01, ***p < 0.001.

To evaluate if dermal exposure to PFHpS was immunosuppressive, the murine IgM response to SRBC was examined following a 10-day exposure to PFHpS. PFHpS significantly reduced specific (PFC/10^6^ cells) and total (PFC/spleen) IgM antibody activity against SRBC at 1.25% (Figure 11(A)) and 1.25 and 2.5% (Figure 11(B)), respectively. Exposure of mice to 1.25% PFHpS resulted in a 41.7% decrease in PFC/10^6^ cells and 2.5% PFHpS exposure in a 62.3% decrease in PFC/spleen vs values for vehicle-treated mice. However, this decrease was not observed in the serum anti-SRBC IgM levels (Figure 11(C)). The significant changes in spleen IgM response to SRBC were dose-dependent and observed in the absence of overt toxicity. PFOS (0.5%) decreased specific IgM antibody activity by 48% and total activity by 60% (Figure 11(A and B)). PFOS also had no effect on the serum anti-SRBC IgM levels (Figure 11(C)). The NK assay was used to evaluate the effect of PFHpS on the innate immune system. A significant increase in NK cell function (measured using a flow-cytometric cytotoxicity assay) was observed with 1.25 and 2.5% PFHpS at 100:1 and 150:1 effector to target ratios and at 150:1 with 0.5% PFOS (Supplemental Figure 2).

Spleen phenotyping was also investigated after dermal exposure to PFHpS for 10 days and immunized with SRBC. PFHpS dermal exposure decreased total cellularity and the number and frequency of eosinophils, neutrophils, and CD11b^+^ cells (Supplemental Fig. 7). Decreases in B-cell and NK cell numbers were observed. CD4^+^ T-cell increases occurred with 1.25 and 2.5% PFHpS. Increased MHC-II MFI was observed on DC while an increase in CD86 MFI was observed on B-cells. PFOS (0.5%) decreased both number and frequency of eosinophils, neutrophils, and CD11b + cells along with increasing MHCII MFI on B-cells and DC and CD86 MFI on B-cells (Supplemental Figure 7).

## Discussion

Dermal exposure is a major occupational concern as the CDC estimates millions of workers in the United States are exposed to chemicals that can be absorbed through the skin (Anderson and Meade 2014; NORA 2019). Data in this study suggest that PFHpS can penetrate mouse skin. PFHpS in the serum and urine increased following a 28-day dermal exposure to 0.3125–1.25% PFHpS. PFOS (0.5%) was detected at a similar concentration to PFHpS in the serum but at a much lower concentration in the urine. Laboratories here have previously investigated several other PFAS compounds, both carboxylic acids (PFBA, PFPeA, PFHxA, PFHpA, PFOA) and sulfonic acids (PFHxS), under the same conditions with similar concentrations. Comparing the 1.25% doses, PFPeA (C5), PFHxA (C6), and PFHpA (C7) all had much lower serum concentrations of 1.86, 1.65, and 62.2 μg/ml, respectively (Weatherly et al. 2023). In comparison, PFHxS (C6) and PFHpS had much higher serum concentrations of 450 and 250 μg/ml, respectively (Weatherly et al. 2024). Inversely, PFPeA (3200 μg/ml), PFHxA (800 μg/ml), and PFHpA (340 μg/ml) showed a much higher concentration in the urine compared to PFHpS (8.1 μg/ml). PFHxS also had a higher concentration in the urine of 43 μg/ml. This data support PFHpS having a longer biological half-life in female mice after dermal exposure compared to carboxylic acid PFAS.

Consistent with previous findings on oral and dermal PFAS exposure in rodents (Shane et al. 2020; ATSDR 2021; Weatherly et al. 2021, 2023, National Toxicology Program [NTP] 2022), one of the main targets of toxicity with dermal PFHpS and PFOS exposure was the liver. Dermal PFHpS and PFOS induced a pronounced increase in liver weight. The large 148% increase in liver weight with 1.25% PFHpS exposure is very similar to the increase seen with 1.25% PFHxS exposure (162%) (Weatherly et al. 2024). Increased liver weight with PFHpS dermal exposure is also consistent with PFHxS oral exposure (Das et al. 2017; Narizzano et al. 2023) and PFOS oral exposure (Wan et al. 2012). Mild (0.3215% PFHpS), moderate (0.625% PFHpS), and marked (1.25% PFHpS) hepatocyte hypertrophy was also observed with PFHpS exposure along with necrosis (3/5 mice with 0.3125%, 5/5 mice with 0.625%, and 5/5 mice with 1.25% PFHpS).

Serum ALKP and ALT, which are hallmark enzymatic markers of liver damage, were increased with PFHpS exposure. Serum increases in these enzymes need to be 2- to 3-fold above control levels for a chemical to be considered as exerting an adverse reaction, according to the US EPA Office of Pesticide Program Guidance Document on Hepatocellular Hypertrophy (US EPA 2002). ALKP increased 2.0- and 2.7-fold with 0.625 and 1.25% PFHpS and ALT increased 4.0- and 4.6-fold with 0.625 and 1.25% PFHpS, respectively. PFOS also increased ALKP (2.4-fold) and ALT (3.8-fold). Based on these results, and recommendations made by previous findings (US EPA 2002; Hall et al. 2012), these data suggest that dermal PFHpS (and dermal PFOS) exposure induced adverse changes in the liver.

Interestingly, PFHpS induced an increase in cholesterol, while PFOS caused a decrease. Previous experiments show that dermal PFHxS also decreases cholesterol, but carboxylic acids do not (Weatherly et al. 2023, 2024). Other studies conducted with oral or dietary PFOS exposure also found decreasing serum cholesterol with increasing PFOS concentration in rodents (Seacat et al. 2003; Martin et al. 2007; Curran et al. 2008). However, many epidemiologic studies show increased cholesterol with higher serum PFOS (Nelson et al. 2010; Fragki et al. 2021). This contrast between animal and human data could be due to differences in dose, serum concentrations, metabolism, and diet. As few studies on PFHpS are available, rodent versus human data is more difficult to compare. However, an epidemiological study did find that higher concentrations of PFHpS were associated with higher levels of cholesterol in humans (Nilsson et al. 2022b).

The only histopathological changes observed in the skin due to PFHpS exposure was epidermal hyperplasia. This is in contrast to dermal carboxylic PFAS exposure which showed that hyperkeratosis, necrosis, inflammation, and fibrosis occurred (Weatherly et al. 2021, 2023). Although there was a significant increase in *Tslp* (with trending increases in *Il-1β* and *S100a8*) and an increase in neutrophil frequency with 2.5%, there was a lack of additional markers that would suggest an inflammatory response. Along with no observed inflammation in histopathology, there was a decrease in eosinophils and *Il-6* in the skin with PFHpS exposure. Further, *Ppar*α—which has been associated with skin inflammation (Furue et al. 2018)—was seen to be decreasing with PFHpS exposure. Therefore, although there is the suggestion of an inflammatory response occurring at the site of exposure, the trends were not consistent, which raises questions about the biological relevance.

No other studies were identified that evaluated the humoral immunity effects of PFHpS. One study with oral PFHxS exposure showed reduction in PFC in deer mice at 7 and 14 mg/kg/day (Narizzano et al. 2023). The laboratories here recently observed a decrease in PFC with dermal PFHxS exposure (Weatherly et al. 2024). The current study shows similar results between dermal PFHpS and PFHxS exposure. Others also saw a decreased SRBC IgM response with PFOS oral exposure, mirroring the current study with dermal PFOS exposure (Peden-Adams et al. 2008; Dong et al. 2009). Although a decrease in IgM PFC was observed with both PFHpS and PFOS, no decrease in serum IgM was seen with either compound. The plaque assay requires coordination between T-cells, B-cells, and macrophages and is considered the “gold standard” for evaluating immunotoxicity (Ladics 2007). It evaluates effects on humoral immunity by measuring splenic cells’ production of antigen-specific antibodies (IgM), while the ELISA measures serum antibody levels of antibodies derived from multiple organs (i.e. spleen, lymph nodes, and bone marrow). In the current study, both assays were conducted 4 days after immunization. In serum, the immune response to SRBC peaks at about 7 days post-immunization (McAllister et al. 2017), so it is possible that PFHpS and PFOS effects on serum IgM were missed due to timing.

NK cells are innate lymphoid cells whose activity can be a measure of nonspecific immunity. Previous studies showed PFOS exposure increased NK-cell function in male mice, but not female mice (Peden-Adams et al. 2008); however, the oral gavage exposure was at much lower concentrations compared to the current study. Another study also saw an increase in NK-cell function in male mice after 83.33 μg/kg/day oral PFOS exposure, similar to the results with dermal PFOS and PFHpS exposure. Interestingly, at higher concentrations of PFOS (833.33 and 2083.33 μg/kg/day) NK-cell function then decreased (Dong et al. 2009). Several other studies found decreased NK-activity with PFOS exposure (Ehrlich et al. 2023). These differences could be due to differing animal models, exposure route, and/or exposure concentration. Ultimately functional immune changes have been reported.

As further evidence of immunotoxicity, PFHpS induced a significant decrease in relative spleen and thymus weight with 1.25% PFHpS. Spleen phenotyping also showed a decrease in total cellularity with 1.25% PFHpS and a significant decrease in B-cell number with 0.625 and 1.25% PFHpS after 28-day exposure. The previously investigated sulfonic acid, PFHxS, also decreased spleen and thymus weight at similar concentrations (Weatherly et al. 2024). However, this trend was not observed with any of the carboxylic acids (Weatherly et al. 2021, 2023). Although NK-cell function increased with PFHpS exposure, NK-cell number decreased with 1.25 and 2.5% PFHpS 10-day exposure and immunization. After a 28-day exposure, PFHpS did not induce a significant change in NK-cell number, although a decreasing trend was observed. Several epidemiological studies with PFAS show immune suppression and other altered immune responses (von Holst et al. 2021). However, very few studies exist on PFHpS and human health compared to other long chain PFAS. The PFHpS effects on immune function in the current study along with the lack of additional studies support the need for continued investigation into immune system effects from PFAS.

Little is known about the importance of dermal uptake as an exposure pathway for PFAS (Ragnarsdóttir et al. 2022). Some studies have suggested that dermal exposure in humans represents only a small portion of total PFAS exposure (Poothong et al. 2020); however, other studies suggest dermal exposure could represent a significant exposure pathway (Carreira et al. 1994; Aas et al. 2014; Thépaut et al. 2021; Lin et al. 2023). A study that developed PBTK models suggested that exposure route does affect PFAS uptake in mice and showed that dermal exposure was the slowest exposure route to peak plasma concentrations. The investigators also showed that dermal exposure exhibited the lowest bioavailability, possibly because of accumulation of PFAS in the skin. It is important to note that PFAS permeability is altered by the pH of the solvent (Franko et al. 2012). Thus, PFAS ionization state needs to be considered when interpreting dermal exposure studies outcomes as cosmetics, firefighting foams, and products associated with other occupations often use different solvents in their PFAS products; as such, the results could be altered PFAS permeability.

It is important to note that mice were group-housed to reduce the potential for unnecessary stress, which can influence immune function. As such, there is the potential for PFAS exposure due to grooming, through the drinking water and food, and tail marking for animal identifycation. These sources could also lead to the detection of PFHpS in control serum. However, these contributions are expected to be minimal and normalized relative to PFHpS exposure. Also, PFHpS levels in exposed groups were 100–250 μg/ml higher compared to control levels. These studies were conducted for hazard identification purposes and to confirm dermal absorption of PFHpS through serum and urine analysis. Therefore, the highest concentrations that showed less than a 10% decrease in weight were selected for evaluation following dermal exposure. Exposure and risk assessment were beyond the scope of these studies and, therefore, not assessed.

Although the detected serum levels in the current study were higher than those seen in the very limited studies with humans, they were comparable to measures in other oral PFAS exposure animal studies (Das et al. 2017; Narizzano et al. 2021, 2023). PFHpS was detected in 98% of adolescents in Norway with a median serum concentration of 0.15 ng/ml (Averina et al. 2018). PFHpS was also detected in 82.6% of the serum samples of adults in Colorado, with a median concentration of 0.2 ng/ml (Barton et al. 2020). PFHpS concentration was found to be higher through occupational exposure compared to in the general public, with a mean concentration of 1.7 ng/ml in serum samples obtained from Australian firefighters (Nilsson et al. 2022a); in another study, professional ski waxers had a median serum concentration of 0.49 ng/ml (Freberg et al. 2010). Higher levels were also detected in communities with PFAS-contaminated drinking water, where the PFHpS median serum concentration was seen to be 12 ng/ml in Nonneby, Sweden (Li et al. 2019). This was consistent with PFAS levels in general which are shown to be higher in occupational exposure versus the general public (Sonnenberg et al. 2023). It should also be noted that comparisons between species are difficult as PFAS are excreted at different rates in different species (ATSDR 2021). In addition, human exposures are often much longer compared to the current study (28-day exposure with mice), and it is possible that in epidemiology studies the peak levels in the serum/urine could be being missed.

These studies are the first to evaluate immunotoxicity induced by PFHpS dermal exposure in a murine model. Significant increases in PFHpS were detected in serum and urine with alterations in organ weights, histology, serum chemistries, gene expression, and SRBC IgM response. These results support that PFHpS can be absorbed through the skin and lead to systemic and immune effects. Further investigation into PFAS dermal exposure is needed to help fill the knowledge gaps regarding the hazards PFAS poses to the immune system.

## Supplementary Material

supplemental material

## Figures and Tables

**Table 1.** Incidence and degree of organ injury following dermal exposure to PFHpS or PFOS in mice.

Parameter	28 days				

	0%	0.3125%	0.625%	1.25%	0.5% PFOS

Liver					
Hypertrophy, hepatocyte					
Mild	0	5	1	0	0
Moderate	0	0	4	0	5
Marked	0	0	0	5	0
Necrosis					
Minimal	0	3	5	5	4
Inflammation, neutrophilic, focal					
Minimal	0	0	2	1	0
Infiltrate, mononuclear cell					
Minimal	5	5	4	2	2
Ear/skin					
Epidermis hyperplasia					
Minimal	0	0	1	1	0
Mild	0	0	0	2	0
Spleen					
Decreased cellularity, lymphocyte					
Minimal	1	2	2	4	3
