# Supplementary material for: Systemic and immunotoxicity induced by topical application of perfluoroheptane sulfonic acid (PFHpS) or perfluorooctane sulfonic acid (PFOS) in a murine model
Source: J Immunotoxicol. Author manuscript; Available in PMC 2024 Dec 1. (PMC11590111; doi:10.1080/1547691X.2024.2371868)
Supplement: supplemental material [file NIHMS2026467-supplement-supplemental_material.docx]

**Supplemental Material**

**Supplemental Table 1. Gating strategies for immunophenotyping**

| **Spleen and dLN cells** |  |
| --- | --- |
| **Cell Type** | **Gating Strategy** |
| CD4 T cells | CD45^+^, lymphocyte gate (FSC/SSC^low^), CD8^-^, B220^-^, NKp46^-^, CD4^+^ |
| CD8 T cells | CD45^+^, lymphocyte gate (FSC/SSC^low^), CD4^-^, B220^-^, NKp46^-^, CD8^+^ |
| B cells | CD45^+^, lymphocyte gate (FSC/SSC^low^), CD4^-^, CD8^-^, NKp46^-^, B220^+^ (MHCII/CD86 MFI increases with activation) |
| NK cells | CD45^+^, lymphocyte gate (FSC/SSC^low^), CD4^-^, CD8^-^, B220^-^, NKp46^+^ |
| Eosinophils | CD45^+^, SSC^high^, CD11b^+^, Ly6G^low^_,_ Siglec-F^+^ |
| Neutrophils | CD45^+^, SSC^high^, CD11b^+^, Siglec-F^-^, Ly6G^high^_,_ |
| DCs | CD45^+^, SSC^int^, MHCII^+^, CD11c^+^, F4/80^-^ (MHCII/CD86 MFI increases with activation) |
| CD11b^+^ monocytes/macrophages | CD45^+^, SSC^int^, CD11b^+^, SiglecF^-^, Ly6G^-^, Lyc6^+/-^, F4/80^+/-^ |
| **Skin (ear) cells** |  |
| **Cell Type** | **Gating Strategy** |
| CD4 T cells | CD45^+^, Lymphocyte gate (FSC/SSC^low^), CD3^+^, CD4^+^, CD8^-^ |
| CD8 T cells | CD45^+^, Lymphocyte gate (FSC/SSC^low^), CD3^+^, CD4^-^, CD8^+^ |
| NK cells | CD45^+^, Lymphocyte gate (FSC/SSC^low^), CD3^-^, NKp46^+^ |
| Eosinophils | CD45^+^, SSC^high^, CD11b^+^, Ly6G^low^_,_ Siglec-F^+^ |
| Neutrophils | CD45^+^, SSC^high^, CD11b^+^, Siglec-F^-^, Ly6G^high^ |
| DCs | CD45^+^, SSC^int^, MHCII^+^, CD11c^+^, F4/80^-^ , CD11b^+/-^ |

**Supplemental Table 2. Genes evaluated in liver, spleen and skin**

| **Gene** | **Assay ID #’s** | **Gene Involvement** |
| --- | --- | --- |
| *Cd36* | Mm00432403_m1 | Steatosis |
| *Lpl* | Mm00434764_m1 | Steatosis |
| *Serpine1* | Mm00435858_m1 | Necrosis |
| *Ehhadh* | Mm00619685_m1 | Fatty Acid Metabolism |
| *Acox1* | Mm00443578_g1 | Fatty Acid Metabolism |
| *Ppara* | Mm00440930_m1 | PPAR Transcription Factor |
| *Il-1beta* | Mm00434228_m1 | Inflammatory Cytokine |
| *Il-6* | Mm00446190_m1 | Inflammatory Cytokine |
| *Tslp* | Mm01157588_m1 | Th2 Skewing Cytokine |
| *Flg2* | Mm02744902_g1 | Skin Barrier |
| *Itgbl1* | Mm01200043_m1 | Skin Barrier |
| *Lor* | Mm01962650_s1 | Skin Barrier |
| *Flg* | Mm01716522_m1 | Skin Barrier |
| *Krt10* | Mm03009921_m1 | Skin Barrier |
| *Krt14* | Mm00516876_m1 | Skin Barrier |
| *S100a8* | Mm00496696_g1 | Danger-Associated Molecular Patterns |
| *Cxcl1* | Mm04207460_m1 | Chemokine |
| *Cpt1b* | Mm00487191_g1 | Fatty Acid Metabolism |
| *Cyp4a10* | Mm02601690_gH | Fatty Acid Metabolism |
| *Pla2g12a* | Mm01316982_m1 | Hepatoxicity |
| *Apoa1* | Mm00437569_m1 | Lipid Transport |
| *Avpr1a* | Mm00444092_m1 | Hepatoxicity |
| *Cyp7a1* | Mm00484150_m1 | Fatty Acid Metabolism |
| *Pparγ* | Mm00440940_m1 | PPAR Transcription Factor |
| *Pparδ* | Mm00803184_m1 | PPAR Transcription Factor |
| *Tlr5* | Mm00546288_s1 | Innate Immunity |
| *Tlr6* | Mm02529782_s1 | Innate Immunity |
| *Tlr7* | Mm04933180_m1 | Innate Immunity |
| *Tlr8* | Mm04209873_m1 | Innate Immunity |
| *Il-10* | Mm01288386_m1 | Anti-inflammatory Cytokine |
| *Crim1* | Mm01232943_m1 | Cytokine/Chemokine Receptor |
| *Ctse* | Mm00456010_m1 | Immunotoxicity |
| *Abcg1* | Mm00843434_s1 | Immunotoxicity |
| *Fabp1* |  | Phospholipidosis |


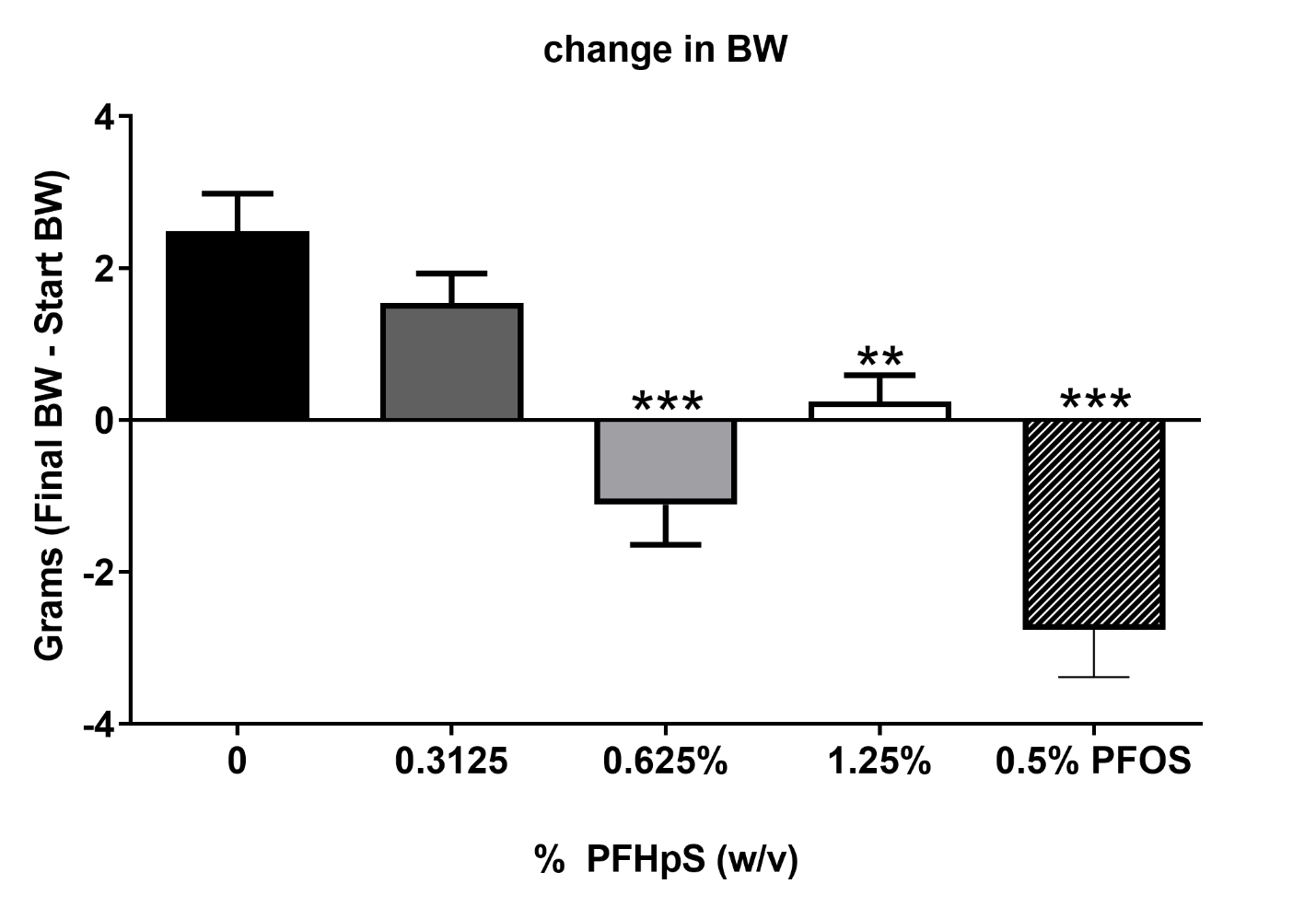


**Supplemental Figure 1. Weight following dermal exposure to PFHpS.** Weight loss (final weight – start weight) following 28-days of PFHpS exposure. Bars represent mean (± SE) of 5 mice per group. Statistical significance, relative to 0% vehicle control, was determined by one-way ANOVA followed by Dunnett’s post-test. Levels of statistical significance are denoted (** p < 0.01, *** p < 0.001) as compared to acetone vehicle (0%).

**Supplemental Table 3. Organ weights following dermal exposure to PFHpS**

|  | **28 Days** | | | | |
| --- | --- | --- | --- | --- | --- |
| PFHpS (w/v) | ***0%*** | ***0.3125%*** | ***0.625%*** | ***1.25%*** | ***0.5% PFOS*** |
| Thymus (g) | 0.045 ± 0.006 | 0.033 ± 0.004 | 0.031 ± 0.007 | **0.013 ± 0.003***** | **0.024 ± 0.006*** |
| Spleen (g) | 0.077 ± 0.007 | 0.070 ± 0.004 | **0.053 ± 0.006*** | **0.047 ± 0.003**** | **0.049 ± 0.005**** |
| Kidneys (g) | 0.312 ± 0.014 | **0.269 ± 0.013*** | **0.246 ± 0.007***** | **0.250 ± 0.006**** | **0.235 ± 0.012**** |
| Liver (g) | 1.153 ± 0.063 | **1.841 ± 0.046***** | **2.187 ± 0.117***** | **2.452 ± 0.105***** | **1.845 ± 0.057***** |

Numbers represent mean (± SE) of 5 mice per group. Levels of statistical significance are denoted (* p < 0.05, ** p < 0.01, and *** p < 0.001) as compared to acetone vehicle (0%).

**
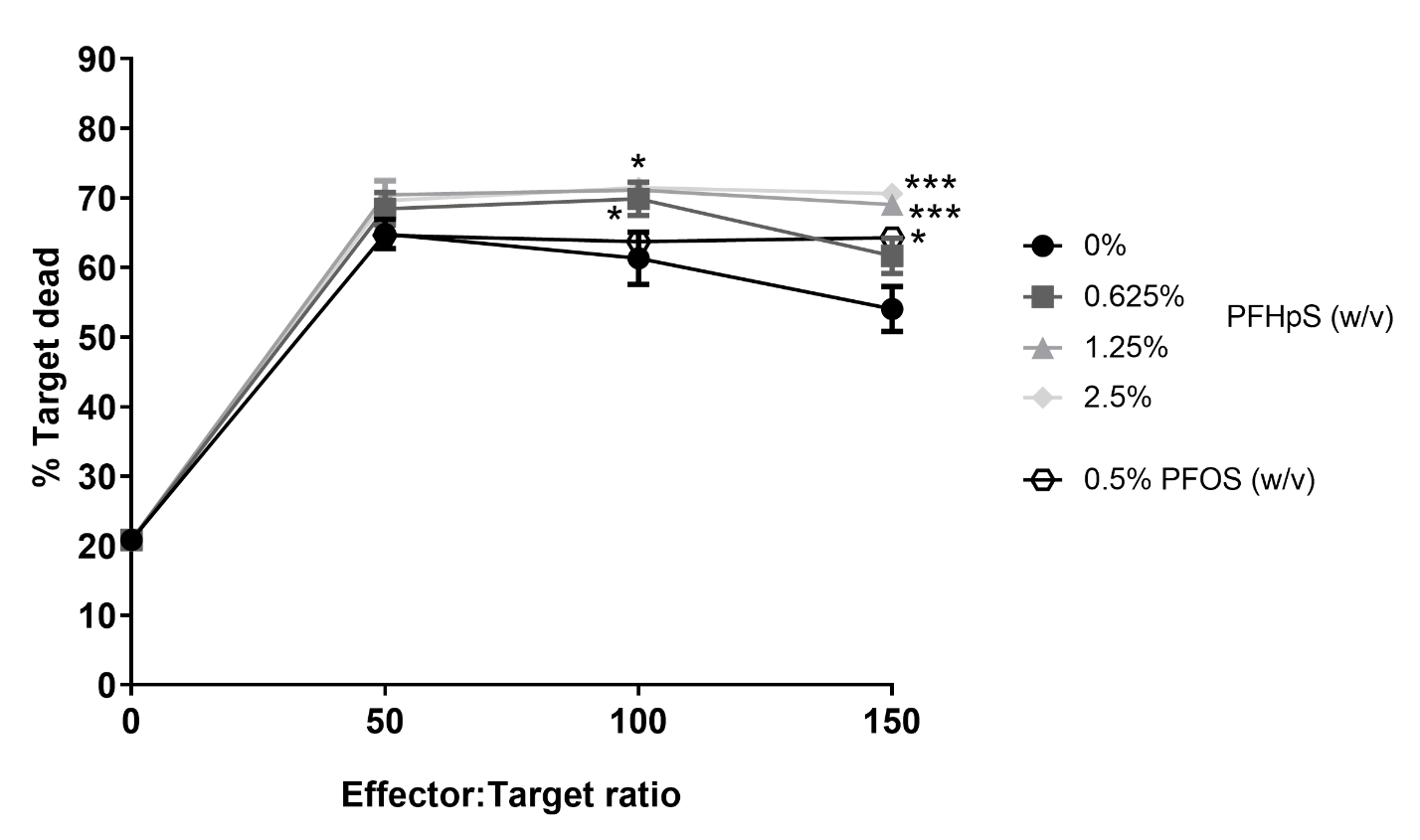
**

**Supplemental Figure 2. Dermal PFHpS exposure alters spleen NK cell activity.** NK cell activity was evaluated in splenocytes after *in vivo* exposure to PFHpS. NK cell percent killing is shown after a 10-day exposure. NK cell activity was evaluated using Yac-1, a murine T-cell lymphoma cell line (ATCC; Manassas, VA) as the target cell. Target cells were cultured in complete media (CM), maintained at 37 ºC in a humidified, 5% CO_2_ atmosphere, and monitored daily. Only the cultures with greater than 95% cell viability were selected for use in the assay. Target cells were labeled with carboxyfluorescein succinmidyl ester (CellTrace^TM^ CFSE Proliferation Kit; Life Technologies; Waltham, MA) according to the manufacturer’s directions. Cells were counted in 0.4% trypan blue solution, and diluted to the desired concentration in CM. Splenocytes collected and processed as described in the Methods section were used as the effector cells. Effector cells were seeded in a 100 µl volume/well with a fixed number of CFSE-stained target cells (20,000/well) with effector to target ratios ranging from 50:1 to 150:1. Control wells for spontaneous death of target contained CFSE-stained target with no effectors (0:1, E:T ratio). Recombinant rat interleukin (IL)-2 (final concentration 0.05 ng/µl; R&D Systems; Minneapolis, MN) was added to well containing effector cells for enhancement of baseline cytotoxic activity. The contents of each well were incubated at 37 °C in a humidified 5% CO_2_ atmosphere for 4 h. All wells were subsequently stained with Live/Dead Fixable Violet Dead Cell Stain and fixed in Cytofix fixation buffer (BD Bioscience) according to the manufacturer’s directions. Effectors only, unstained target, and Live/Dead Fixable Violet Dead Cell (Invitrogen; Waltham, MA)-stained targets were also included as assay controls. Stained samples were analyzed on a LSR II flow cytometer (BD Biosciences). The results were expressed as percentage of dead targets on a cell-to-cell basis. Data shown as the means ± SEM of 5 mice per group. Statistical significance, relative to 0% control, was determined by one-way ANOVA followed by Dunnett’s post-test (PFHpS) or a t-test (PFOS) indicated as *p < 0.05, ***p < 0.001.

**Supplemental Table 4.** Skin phenotyping of mice dermally exposed to PFHpS or PFOS

| **Skin** | **28 days** | | | | |
| --- | --- | --- | --- | --- | --- |
| **Parameter** | **0%** | **0.3125%** | **0.625%** | **1.25%** | **0.5% PFOS** |
| Cellularity (x 10^6^) | 3.06 ± 0.20 | **2.37 ± 0.08*** | 2.55 ± 0.16 | **2.05 ± 0.21**** | **1.75 ± 0.07***** |
| CD45+ (x 10^5^) | 2.53 ± 0.28 | 2.21 ± 0.14 | 2.14 ± 0.18 | 1.77 ± 0.30 | **1.35 ± 0.05**** |
| CD45+ (%) | 8.21 ± 0.57 | 9.29 ± 0.41 | 8.36 ± 0.28 | 8.35 ± 0.90 | 7.72 ± 0.26 |
| CD4+ (x 10^3^) | 4.73 ± 0.58 | 3.83 ± 0.46 | 4.18 ± 0.32 | 4.31 ± 0.90 | **2.97 ± 0.32*** |
| CD4+ (%) | 1.89 ± 0.19 | 1.71 ± 0.11 | 1.97 ± 0.12 | 2.41 ± 0.27 | 2.18 ± 0.18 |
| CD8+ (x 10^2^) | 4.47 ± 0.38 | 3.89 ± 0.29 | 4.77 ± 0.60 | 5.74 ± 1.26 | 9.32 ± 2.09 |
| CD8+ (%) | 0.18 ± 0.01 | 0.18 ± 0.01 | 0.22 ± 0.02 | **0.31 ± 0.05**** | **0.67 ± 0.13**** |
| NK (x 10^3^) | 8.10 ± 1.18 | 7.20 ± 1.01 | 6.23 ± 1.05 | 4.65 ± 1.05 | **3.13 ± 0.31**** |
| NK (%) | 3.17 ± 0.18 | 3.23 ± 0.33 | 2.85 ± 0.28 | 2.42 ± 0.32 | **2.30 ± 0.18**** |
| Eosinophils (x 10^3^) | 5.83 ± 0.75 | **2.72 ± 0.50**** | **2.94 ± 0.42***** | **1.05 ± 0.19***** | **2.84 ± 0.93*** |
| Eosinophils (%) | 2.30 ± 0.09 | **1.23 ± 0.20***** | **1.35 ± 0.10***** | **0.61 ± 0.06***** | 2.04 ± 0.64 |
| Neutrophils (x 10^2^) | 9.67 ± 1.65 | 5.89 ± 0.48 | 6.72 ± 0.24 | 25.00 ± 8.64 | 6.96 ± 0.77 |
| Neutrophils (%) | 0.39 ± 0.07 | 0.27 ± 0.02 | 0.32 ± 0.02 | **1.29 ± 0.39*** | 0.51 ± 0.04 |
| CD11b- DCs (x 10^3^) | 6.03 ± 1.19 | 5.32 ± 0.52 | 4.53 ± 0.46 | **1.73 ± 0.34**** | **2.19 ± 0.29*** |
| CD11b- DCs (%) | 2.31 ± 0.23 | 2.41 ± 0.16 | 2.11 ± 0.14 | **1.06 ± 0.19***** | **1.61 ± 0.18*** |
| CD11b+ DCs (x 10^4^) | 1.31 ± 0.14 | 1.11 ± 0.08 | 0.95 ± 0.09 | 1.04 ± 0.23 | **0.69 ± 0.08**** |
| CD11b+ DCs (%) | 5.23 ± 0.30 | 5.09 ± 0.39 | 4.41 ± 0.26 | 5.49 ± 0.60 | 5.09 ± 0.42 |

Values are expressed as the means (± SE) for each group (n=5 mice/group). Cell numbers and frequency are per one ear.

Dunnett’s post-test (PFHpS) or a t-test (PFOS) indicated as *p < 0.05, **p<0.01, ***p<0.001

**Supplemental Table 5.** Draining lymph node phenotyping of mice dermally exposed to PFHpS

| **dLN** | **28 days** | | | | | |
| --- | --- | --- | --- | --- | --- | --- |
| **Parameter^a^** | **0%** | **0.3125%** | **0.625%** | **1.25%** | **0.5% PFOS** |  |
| Cellularity (x 10^6^) | 3.92 ± 0.69 | 5.28 ± 0.69 | 3.61 ± 0.76 | 4.86 ± 0.96 | 3.73 ± 0.63 |  |
| CD4+ (x 10^6^) | 1.50 ± 0.26 | 2.17 ± 0.29 | 1.38 ± 0.31 | 1.65 ± 0.34 | 1.34 ± 0.26 |  |
| CD4+ (%) | 37.58 ± 2.00 | 41.02 ± 0.77 | 39.02 ± 1.57 | 33.82 ± 0.44 | 35.18 ± 1.29 |  |
| CD8+ (x 10^6^) | 1.10 ± 0.20 | 1.61 ± 0.21 | 1.16 ± 0.21 | 1.66 ± 0.31 | 1.10 ± 0.19 |  |
| CD8+ (%) | 27.38 ± 1.60 | 30.52 ± 0.66 | **32.28 ± 1.22*** | **34.58 ± 0.93***** | 29.30 ± 0.54 |  |
| B-cells (x 10^5^) | 8.55 ± 1.52 | 9.24 ± 1.27 | 5.51 ± 1.65 | 9.46 ± 2.10 | 7.17 ± 1.20 |  |
| B-cells (%) | 22.92 ± 3.12 | 17.48 ± 0.35 | **14.26 ± 1.30*** | 19.20 ± 0.88 | 19.22 ± 0.35 |  |
| NK (x 10^3^) | 8.17 ± 0.62 | 9.16 ± 1.94 | 8.19 ± 1.29 | 7.38 ± 1.73 | 8.13 ± 0.46 |  |
| NK (%) | 0.23 ± 0.03 | 0.18 ± 0.03 | 0.31 ± 0.07 | 0.15 ± 0.01 | 0.25 ± 0.06 |  |
| Eosinophils (x 10^3^) | 2.30 ± 0.50 | 1.84 ± 0.38 | 1.83 ± 0.78 | 2.10 ± 0.51 | 5.14 ± 1.57 |  |
| Eosinophils (%) | 0.06 ± 0.01 | 0.03 ± 0.01 | 0.05 ± 0.01 | 0.04 ± 0.01 | **0.12 ± 0.03*** |  |
| Neutrophils (x 10^3^) | 3.18 ± 0.66 | 2.72 ± 0.47 | 2.36 ± 0.35 | 2.83 ± 0.45 | 3.98 ± 0.85 |  |
| Neutrophils (%) | 0.10 ± 0.04 | 0.05 ± 0.01 | 0.06 ± 0.01 | 0.07 ± 0.02 | 0.12 ± 0.03 |  |
| Dendritic Cells (x 10^4^) | 4.09 ± 0.85 | 5.98 ± 0.60 | 6.49 ± 1.08 | **10.90 ± 1.75***** | 6.95 ± 0.55 |  |
| Dendritic Cells (%) | 1.02 ± 0.11 | 1.18 ± 0.12 | 1.83 ± 0.23 | **2.40 ± 0.32**** | **2.14 ± 0.45*** |  |
| CD11b+ (x 10^4^) | 6.35 ± 1.00 | 7.95 ± 1.37 | 5.95 ± 1.27 | 9.05 ± 1.74 | 7.63 ± 0.98 |  |
| CD11b+ (%) | 1.62 ± 0.05 | 1.51 ± 0.16 | 1.87 ± 0.21 | 1.93 ± 0.25 | 2.14 ± 0.16 |  |
| MHCII B-cells (MFI) | 1098.80 ± 78.95 | 1335.40 ± 54.97 | 1581.40 ± 431.59 | **1878.40 ± 136.47***** | 1296.60 ± 63.62 |  |
| MHCII DCs (MFI) | 7154.00 ± 903.45 | 7977.60 ± 484.42 | 7125.40 ± 607.36 | **12843.80 ± 893.22***** | 5635.40 ± 651.94 |  |
| CD86 B-cells (MFI) | 320.60 ± 7.85 | 408.00 ± 7.49 | 505.40 ± 84.48 | **476.60 ± 31.39*** | 423.20 ± 40.17 |  |
| CD86 DCs (MFI) | 1879.00 ± 169.81 | 2248.60 ± 105.03 | 2278.60 ± 410.40 | **3423.80 ± 151.79***** | 1688.00 ± 126.87 |  |

Values are expressed as the means (± SE) for each group (n=5 mice/group). Cell numbers and frequency are per two draining lymph nodes.

*p < 0.05, **p<0.01, ***p<0.001

**Supplemental Table 6.** Spleen phenotyping of mice dermally exposed to PFHpS or PFOS

| **Spleen** | **28 days** | | | | |
| --- | --- | --- | --- | --- | --- |
| **Parameter** | **0%** | **0.3125%** | **0.625%** | **1.25%** | **0.5% PFOS** |
| Cellularity (x 10^7^) | 11.30 ± 1.74 | 9.02 ± 1.37 | 7.08 ± 1.53 | **5.01 ± 0.74*** | **5.82 ± 0.85*** |
| CD4+ (x 10^7^) | 2.13 ± 0.33 | 1.86 ± 0.23 | 1.62 ± 0.37 | 1.12 ± 0.14 | 1.51 ± 0.21 |
| CD4+ (%) | 18.70 ± 0.69 | 21.10 ± 1.13 | **22.70 ± 1.42*** | **22.52 ± 0.66*** | **26.10 ± 0.42***** |
| CD8+ (x 10^6^) | 13.20 ± 2.28 | 11.60 ± 1.28 | 8.62 ± 2.04 | 7.51 ± 1.12 | 8.82 ± 0.93 |
| CD8+ (%) | 11.50 ± 0.65 | 13.36 ± 0.96 | 11.95 ± 1.05 | **15.00 ± 0.18*** | **15.70 ± 0.98**** |
| B-cells (x 10^7^) | 4.55 ± 0.73 | 3.48 ± 0.65 | **2.53 ± 0.55*** | **1.80 ± 0.23**** | **2.02 ± 0.32**** |
| B-cells (%) | 40.28 ± 1.34 | 38.00 ± 1.72 | 35.90 ± 1.13 | 36.24 ± 0.80 | **34.36 ± 0.97**** |
| NK (x 10^5^) | 8.56 ± 1.54 | 9.31 ± 2.12 | 5.43 ± 0.95 | 4.18 ± 0.81 | 4.92 ± 0.80 |
| NK (%) | 0.76 ± 0.13 | 1.02 ± 0.11 | 0.79 ± 0.04 | 0.82 ± 0.05 | 0.85 ± 0.05 |
| Neutrophils (x 10^5^) | 12.10 ± 2.13 | 7.75 ± 1.80 | 6.81 ± 1.50 | 5.99 ± 1.25 | **5.39 ± 0.70*** |
| Neutrophils (%) | 1.06 ± 0.11 | 0.83 ± 0.09 | 0.97 ± 0.08 | 1.18 ± 0.11 | 0.98 ± 0.14 |
| Dendritic Cells (x 10^6^) | 4.92 ± 0.78 | 4.44 ± 0.81 | 3.26 ± 0.85 | 2.41 ± 0.51 | **2.27 ± 0.36*** |
| Dendritic Cells (%) | 4.29 ± 0.12 | 4.82 ± 0.24 | 4.50 ± 0.21 | 4.70 ± 0.36 | **3.88 ± 0.12*** |
| CD11b+ (x 10^6^) | 3.06 ± 0.44 | 2.28 ± 0.34 | **1.51 ± 0.38*** | **1.16 ± 0.18**** | **1.26 ± 0.27**** |
| CD11b+ (%) | 2.74 ± 0.26 | 2.54 ± 0.07 | **2.09 ± 0.09*** | 2.30 ± 0.07 | 2.09 ± 0.22 |
| CD11b+Ly6C+ (x 10^5^) | 5.43 ± 0.91 | 3.78 ± 0.54 | 2.85 ± 0.71 | 2.95 ± 0.60 | **2.60 ± 0.32*** |
| CD11b+Ly6C+ (%) | 0.48 ± 0.05 | 0.42 ± 0.02 | 0.40 ± 0.02 | 0.58 ± 0.05 | 0.46 ± 0.02 |
| CD11b+Ly6C- (x 10^5^) | 25.10 ± 3.47 | 17.00 ± 2.66 | **12.20 ± 3.09*** | **8.60 ± 1.28**** | **10.00 ± 2.37**** |
| CD11b+Ly6C- (%) | 2.26 ± 0.21 | 1.95 ± 0.21 | 1.70 ± 0.07 | 1.72 ± 0.03 | 1.64 ± 0.22 |
| MHCII B-cells (MFI) | 935.40 ± 41.20 | 1016.80 ± 16.46 | 958.00 ± 48.31 | **1120.20 ± 32.77**** | 936.00 ± 65.53 |
| MHCII DCs (MFI) | 1937.80 ± 74.70 | 1915.20 ± 60.75 | 2044.80 ± 69.75 | 2144.00 ± 122.97 | 2078.20 ± 56.58 |
| CD86 B-cells (MFI) | 275.20 ± 11.73 | **333.20 ± 17.96*** | 309.00 ± 11.35 | 317.00 ± 11.35 | 241.20 ± 10.32 |
| CD86 DCs (MFI) | 1570.20 ± 65.93 | 1565.60 ± 72.92 | **1244.80 ± 74.04*** | 1509.80 ± 82.74 | **1364.60 ± 22.87*** |

Values are expressed as the means (± SE) for each group (n=5 mice/group). Cell numbers and frequency are cell number per spleen.

Dunnett’s post-test (PFHpS) or a t-test (PFOS) indicated as *p < 0.05, **p<0.01, ***p<0.001

**Supplemental Table 7.** Spleen phenotyping of mice dermally exposed to PFHpS for 10-days and immunized with SRBC

| **Spleen** | **28 days** | | | | |
| --- | --- | --- | --- | --- | --- |
| **Parameter** | **0%** | **0.625%** | **1.25%** | **2.5%** | **0.5% PFOS** |
| Cellularity (x 10^7^) | 23.10 ± 2.23 | 20.60 ± 3.29 | 16.20 ± 1.06 | **14.70 ± 2.31*** | 17.70 ± 1.62 |
| CD4+ (x 10^7^) | 4.86 ± 0.58 | 4.63 ± 0.83 | 3.97 ± 0.26 | 3.69 ± 0.53 | 3.93 ± 0.40 |
| CD4+ (%) | 20.88 ± 1.05 | 22.18 ± 0.69 | **24.56 ± 0.62**** | **25.28 ± 0.35**** | 22.12 ± 0.65 |
| CD8+ (x 10^7^) | 2.88 ± 0.32 | 2.58 ± 0.41 | 2.10 ± 0.16 | 1.91 ± 0.29 | 2.19 ± 0.21 |
| CD8+ (%) | 12.38 ± 0.34 | 12.50 ± 0.22 | 12.94 ± 0.24 | 13.08 ± 0.32 | 12.38 ± 0.15 |
| B-cells (x 10^7^) | 8.46 ± 0.64 | 7.66 ± 1.27 | 5.97 ± 0.46 | **5.08 ± 0.93*** | 7.01 ± 0.63 |
| B-cells (%) | 36.96 ± 1.46 | 37.00 ± 0.82 | 36.76 ± 0.97 | 34.02 ± 1.11 | 39.74 ± 1.14 |
| NK (x 10^6^) | 5.45 ± 0.56 | 4.86 ± 0.54 | **3.54 ± 0.23*** | **3.08 ± 0.59*** | 3.80 ± 0.48 |
| NK (%) | 2.35 ± 0.09 | 2.43 ± 0.12 | 2.19 ± 0.08 | 2.06 ± 0.09 | 2.13 ± 0.14 |
| Eosinophils (x 10^5^) | 7.00 ± 0.89 | 6.39 ± 1.24 | **3.34 ± 0.14*** | **2.19 ± 0.36**** | **3.37 ± 0.31**** |
| Eosinophils (%) | 0.30 ± 0.02 | 0.31 ± 0.04 | 0.21 ± 0.01 | **0.15 ± 0.01**** | **0.19 ± 0.01**** |
| Neutrophils (x 10^6^) | 3.19 ± 0.38 | **2.22 ± 0.23*** | **1.40 ± 0.96***** | **1.36 ± 0.23***** | **1.08 ± 0.08***** |
| Neutrophils (%) | 1.37 ± 0.07 | **1.12 ± 0.08*** | **0.86 ± 0.02***** | **0.93 ± 0.07***** | **0.62 ± 0.05***** |
| Dendritic Cells (x 10^6^) | 5.52 ± 0.59 | 5.61 ± 0.95 | 5.09 ± 0.41 | 5.56 ± 1.29 | 5.54 ± 0.67 |
| Dendritic Cells (%) | 2.38 ± 0.07 | 2.73 ± 0.37 | 3.14 ± 0.10 | **3.62 ± 0.32**** | **3.13 ± 0.21*** |
| CD11b+ (x 10^6^) | 5.20 ± 0.65 | 4.15 ± 0.52 | **2.56 ± 0.24**** | **2.23 ± 0.21***** | **2.53 ± 0.23**** |
| CD11b+ (%) | 2.23 ± 0.12 | 2.09 ± 0.18 | **1.60 ± 0.16*** | **1.59 ± 0.12*** | **1.44 ± 0.06***** |
| CD11b+Ly6C+ (x 10^5^) | 16.20 ± 2.21 | 13.10 ± 1.86 | **10.00 ± 0.69*** | **7.57 ± 1.10**** | **8.16 ± 0.75**** |
| CD11b+Ly6C+ (%) | 0.69 ± 0.06 | 0.64 ± 0.02 | 0.62 ± 0.04 | **0.52 ± 0.05*** | **0.46 ± 0.02**** |
| CD11b+Ly6C- (x 10^5^) | 32.30 ± 4.07 | 25.40 ± 3.12 | **13.60 ± 1.18***** | **13.20 ±1.18***** | **15.20 ± 1.60**** |
| CD11b+Ly6C- (%) | 1.38 ± 0.06 | 1.29 ± 0.14 | **0.85 ± 0.11*** | **0.95 ± 0.12*** | **0.86 ± 0.06***** |
| MHCII B-cells (MFI) | 2278.40 ± 56.78 | 2303.60 ± 268.29 | 2321.80 ± 66.27 | 1933.00 ± 75.26 | **2668.60 ± 104.91*** |
| MHCII DCs (MFI) | 2963.60 ± 47.12 | 3152.60 ± 53.92 | **3320.80 ± 94.96*** | **3629.00 ± 105.58***** | **3177.40 ± 45.02*** |
| CD86 B-cells (MFI) | 98.30 ± 1.19 | 103.36 ± 5.69 | **116.80 ± 2.84*** | **121.40 ± 4.21***** | **138.60 ± 3.43***** |
| CD86 DCs (MFI) | 628.80 ± 38.96 | 710.00 ± 48.22 | 640.20 ± 32.49 | **785.60 ± 30.20*** | 593.00 ± 19.27 |

Values are expressed as the means (± SE) for each group (n=5 mice/group).

*p < 0.05, **p<0.01, ***p<0.001
